# Catalpol mitigates rheumatoid arthritis by targeting neutrophil extracellular trap release

**DOI:** 10.3389/fimmu.2026.1763586

**Published:** 2026-03-16

**Authors:** Chaoding Li, Liping Wang, Qianyu Li, Wenjie Zhao, Zhengguang Hui, Zhen Zhang, Lu Zhao

**Affiliations:** 1 Department of Orthopedics, Xuzhou Hospital of Traditional Chinese Medicine, Xuzhou, China; 2 Jiangsu Key Laboratory of New Drug Research and Clinical Pharmacy, Xuzhou Medical University, Xuzhou, China

**Keywords:** cartilage degradation, catalpol, neutrophil extracellular traps, PAD4, rheumatoid arthritis

## Abstract

**Background:**

Rheumatoid arthritis (RA) is a chronic autoimmune disorder characterized by persistent synovial inflammation, progressive joint damage, and systemic manifestations. Current therapies, including NSAIDs, DMARDs, and biologics, face limitations such as side effects and high costs. Neutrophil extracellular traps (NETs), driven by peptidylarginine deiminase 4 (PAD4), play a pivotal role in RA pathogenesis by promoting inflammation and cartilage degradation. Catalpol (CAT), an iridoid glycoside known for its anti-inflammatory properties, exhibits therapeutic promise through targeting NET-related mechanisms.

**Methods:**

A collagen-induced arthritis (CIA) model was established in male DBA/1 mice. Mice were treated with CAT (30 mg/kg) or vehicle. Joint damage was assessed via micro-CT and histological staining (H&E, Safranin O, Toluidine Blue). NETs and inflammatory markers were analyzed by immunohistochemistry, Western blot, qRT-PCR, and immunofluorescence. Isolated neutrophils were stimulated with PMA *in vitro* to study CAT’s direct effects on NETosis. Transcriptomic analysis was performed on joint tissues to identify potential targets.

**Results:**

CAT treatment significantly ameliorated ankle swelling, bone erosion, synovial hyperplasia, inflammatory cell infiltration, and cartilage degradation in CIA mice. It suppressed the expression of NET-associated markers (Cit-H3, MPO, NE) and pro-inflammatory cytokines (IL-1β, IL-6) in joint tissues. CAT also inhibited the expression of matrix-degrading enzymes (MMP3, MMP13) and osteoclast-related factors (MMP9, CTSK, Tracp-5b). *In vitro*, CAT (10 μM) effectively inhibited PMA-induced NET formation in primary neutrophils. Furthermore, CAT reduced NET-induced ROS production, mitochondrial membrane potential loss, and apoptosis in chondrocytes. Mechanistically, transcriptomic analysis suggested enrichment in neutrophil-related pathways, and Western blot confirmed that CAT inhibited PAD4 protein expression, a key regulator of NETosis.

**Conclusion:**

Catalpol exerts a protective effect in the CIA model by inhibiting PAD4-mediated NET formation, thereby reducing synovial inflammation, cartilage degradation, and bone erosion. Our findings highlight NETs as a promising therapeutic target and suggest catalpol’s potential as a novel agent for RA treatment.

## 1 Introduction

Rheumatoid arthritis (RA) is a chronic, inflammatory, multisystemic autoimmune disorder of unknown origin, primarily affecting the peripheral joints in a symmetrical manner (1). It is characterized by the production of autoantibodies targeting citrullinated peptides, cytokine imbalances, joint inflammation, synovial hyperplasia, and the invasion of the synovium into the adjacent bone and cartilage, resulting in progressive joint destruction (2). RA is associated with long-term disability, premature mortality, and substantial socioeconomic burdens. The global prevalence of RA varies widely, ranging from 0.25% to 1%. Although RA can affect individuals of any age, its incidence increases among those over 40 years old (3). While significant progress has been made in understanding the pathophysiology of RA, its exact etiology remains elusive.

Pharmacological management of RA encompasses non-steroidal anti-inflammatory drugs (NSAIDs), glucocorticoids (GCs), conventional synthetic disease-modifying antirheumatic drugs (csDMARDs), and biologic agents. NSAIDs and GCs primarily alleviate acute symptoms but do not modify disease progression, with long-term use limited by gastrointestinal/cardiovascular risks and metabolic/infectious complications, respectively (4, 5). csDMARDs such as methotrexate exhibit delayed onset and variable efficacy, often accompanied by hepatotoxicity and hematological adverse effects6. Although biologic therapies provide targeted immunomodulation and improve outcomes, their utility is constrained by infection risks, immunogenicity, and high costs (6). Collectively, existing treatments ameliorate symptoms without reversing established pathology, underscoring the need for novel agents that fundamentally disrupt disease mechanisms. (7, 8). Therefore, identifying new drug targets and developing therapies that can effectively suppress the pathological processes, reduce inflammation, and promote tissue repair have become key directions for ongoing research.

The role of Neutrophil Extracellular Traps (NETs) in disease development and as a drug target has garnered increasing attention in recent years. Specifically, NETs play a crucial role in the pathogenesis of RA, particularly in inflammation and cartilage damage (7–9). NETs are mesh-like structures actively released by neutrophils through a programmed cell death pathway called NETosis. Their core structure consists of DNA, core histones, and various granule proteins. Abnormal formation of NETs in RA is considered a key factor driving disease progression (10). In RA patients, NET formation is significantly increased, primarily driven by Peptidylarginate Deiminase 4 (PAD4). PAD4, a calcium-dependent enzyme, catalyzes the citrullination of histone arginine residues, leading to chromatin decondensation and ultimately driving NET release. In RA, PAD4 directly contributes to synovial inflammation and the amplification of autoimmune responses by mediating NET formation and the release of pro-inflammatory cytokines (11, 12). The formation of NETs may be triggered by various factors, including immune complex deposition, pro-inflammatory cytokines like IL-1, IL-6, and TNF-α, and direct stimulation by Anti-Citrullinated Protein Antibodies (ACPA) (13, 14). In cartilage damage, NETs release proteases and pro-inflammatory mediators that directly degrade cartilage matrix components, leading to cartilage destruction and joint dysfunction. Studies have shown that Neutrophil Elastase (NE) and Myeloperoxidase (MPO) in NETs can degrade collagen and proteoglycans in the cartilage matrix, disrupting its structural integrity. Additionally, NETs activate the expression of Matrix Metalloproteinases (MMPs) and the ADAMTS family genes, further exacerbating the breakdown of the cartilage matrix (15–17). Furthermore, proteases in NETs not only directly degrade cartilage but also accumulate in joint areas, attracting macrophages and osteoclasts. Activated macrophages release pro-inflammatory cytokines such as TNF-α and IL-6, which not only intensify local inflammation but also promote osteoclast differentiation and activation. Osteoclasts, in turn, secrete factors like RANKL and Tracp-5b, directly participating in the degradation of bone matrix (18–20).

Catalpol (CAT) is a significant bioactive compound in the class of iridoid glycosides, widely found in traditional Chinese medicinal herbs such as Rehmannia. It exhibits a variety of pharmacological effects, including anti-inflammatory, antioxidant, immune-modulatory, neuroprotective, and anti-tumor activities (21, 22). Studies have shown that in osteoarthritis (OA), CAT can prevent IL-1β-induced chondrocyte apoptosis and effectively inhibit the secretion and release of matrix metalloproteinases. Additionally, catalpol alleviates extracellular matrix degradation mediated by IL-1β. Moreover, CAT reduces the secretion of inflammatory factors such as IL-6 and TNF-α by blocking the NF-κB signaling pathway, thereby mitigating the inflammatory response. In animal models, CAT has been shown to effectively improve the progression of knee osteoarthritis cartilage degeneration in OA models, demonstrating its potential therapeutic efficacy in treating osteoarthritis by alleviating inflammation and suppressing metabolic processes (23). Research has suggested that CAT reduces synovial inflammation by inhibiting the NF-κB signaling pathway, regulates the balance between Th17 and Treg cells to restore immune homeostasis, and protects joint structure by inhibiting osteoclast activity (24, 25).

While catalpol’s known anti-inflammatory and immunomodulatory effects likely contribute to its benefits in RA models, its potential impact on NETosis, a key amplifier of synovitis and joint damage, remains unexplored. Given that inflammatory pathways like NF-κB can regulate PAD4 expression, a key enzyme in NET formation, we hypothesized that catalpol might protect against RA in part by suppressing PAD4-dependent NETosis. The collagen-induced arthritis (CIA) model, a widely adopted experimental paradigm for RA research, likely incorporates PAD4-dependent NET formation in its pathogenic mechanism. Specifically, autoantibodies directed against PAD4 or citrullinated proteins, including anti-citrullinated protein antibodies, can activate PAD4, thereby inducing NET release. Subsequent exposure of citrullinated autoantigens may then propagate autoimmune responses, collectively driving arthritis initiation and progression in the CIA model (26, 27).

Thus, this compound has the potential to exert anti- RA effects through inhibiting the formation of Neutrophil Extracellular Traps (NETs), although its specific mechanism of action remains unclear. This study will explore its mechanism in both animal and cell models.

## 2 Methods and materials

### 2.1 Reagents

The principal reagents and kits employed in this study are listed as follows. Immunization Grade Bovine Type II Collagen and Complete Freund’s Adjuvant were obtained from Chondrex, Inc. Catalpol (HPLC ≥ 98%) was purchased from Nanjing Jingzhu Bioengineering Co., Ltd (Cat# Z100599). RIPA Lysis Buffer (Strong; Cat# P0013B), PMSF (100 mM; Cat# ST506), 5×SDS-PAGE Loading Buffer (Cat# P0015), Penicillin-Streptomycin Solution (100×; Cat# C0222), Mitochondrial Membrane Potential and Apoptosis Assay Kit (Cat# C1071M), and Antifade Mounting Medium with DAPI (Cat# P0131) were acquired from Beyotime Biotechnology. The One-Step PAGE Gel Fast Preparation Kit (Cat# E304-01) was provided by Nanjing Vazyme Biotech Co., Ltd. Primary antibodies against IL-6 (Cat# 66146-1-lg), IL-1β (Cat# 26048-1-AP), MMP3 (Cat# 17873-1-AP), MMP13 (Cat# 118165-1-AP), and NE (Cat# AF0010) were obtained from Proteintech. Anti-Cit-H3 antibody (Cat# AB281584) was from Abcam, and anti-MPO antibody (Cat# EM1901-19) was procured from HuaAn Biotechnology. For detection, HRP-conjugated secondary antibodies including Anti-Rabbit IgG (Cat# BS13278; Bioworld Technology) and Anti-Mouse IgG (Cat# SA00001-1; Proteintech) were used. Fluorescent secondary antibodies, namely Dlight 488-goat anti-Rabbit (Cat# BS10017) and Dlight 594-goat anti-Mouse (Cat# BS10027), were supplied by Bioworld Technology, while Dlight 594-goat anti-rat IgG (Cat# E032440-01) was from EarthOx. Other essential reagents included the DAB Detection Kit (Cat# ZLI-9018) and the Ultrasensitive™ Polymer Detection Kit (for Mouse/Rabbit; Cat# PV-8000) from ZSGB-BIO, the ECL Substrate Kit (Cat# A0409A01) from Absin Bioscience Inc., the Mouse Bone Marrow Neutrophil Extraction Kit (Cat# P8550) from Solarbio, and the Reactive Oxygen Species Assay Kit (Cat# BL714A) from Biosharp Life Sciences. Cell culture reagents, including DMEM (Cat# C11995500BT) and Protein Marker (Cat# 26616), were purchased from Thermo Fisher Scientific, and Fetal Bovine Serum (Cat# C04001-100) was obtained from VivaCell.

### 2.2 Ethics statement

The present study obtained approval from the Animal Ethics Committee of Xuzhou Medical University (Xuzhou, China, No. 202307T008). Efforts were made to minimize pain and discomfort in the animals. All animal experiments were conducted at Xuzhou Medical University.

### 2.3 Animals

Male DBA/1 mice (7 weeks old, weighing 20 ± 2 g) were obtained from the Laboratory Animal Center of Xuzhou Medical University (Xuzhou, China), were selected for this study due to their well-established and reproducible susceptibility to collagen-induced arthritis (CIA), thus providing a stable model system for initial mechanistic investigation while minimizing potential confounders related to sex-specific immune variability during the proof-of-concept evaluation of catalpol’s effects.

### 2.4 Establishment of the CIA mouse model

After a one-week acclimatization period, mice were randomly divided into three groups: Normal group (N, n=6), Rheumatoid Arthritis group (CIA, n=6), and CIA + Treatment group (CIA+CAT, n=6). An emulsion containing 0.1 mL (100 mg collagen) was injected into the base of the tail of each mouse. The Normal group received the same volume of saline at the same site. The first injection was considered Day 0. On Day 21, an emulsion was prepared by thoroughly emulsifying an equal volume of bovine type II collagen with incomplete Freund’s adjuvant. This emulsion was then injected subcutaneously into the base of the mouse tail (0.1 mL) for booster immunization. From Day 28 onwards, the following treatment regimen was applied to each group of mice: Normal Group (N): Oral administration of saline every 3 days for 4 weeks. CIA Group (Rheumatoid Arthritis Model): Oral administration of saline every 3 days for 4 weeks. CIA + Treatment Group (CIA+CAT): Oral administration of CAT solution (30 mg/kg) every 3 days for 4 weeks. Catalpol (30 mg/kg) or vehicle was administered intraperitoneally daily from day 21 post-immunization, representing a preventive intervention regimen. After 4 weeks of treatment, mice were euthanized, and their ankle joints were harvested. Some specimens were frozen, while others were fixed for further analysis. Micro-CT scanning, H&E staining, Toluidine Blue staining, and Safranin O-fast green staining were performed to assess the pathological changes and extent of cartilage damage in the ankle joints.

### 2.5 Micro-CT scanning

The right hind limb of DBA/1 male mice was selected for the study. Scanning was performed using the VENUS Micro-CT *in vivo* imaging system with the following parameters: energy set to 90 kV and current at 0.07 mA. Three-dimensional reconstruction was performed using the built-in Cruiser software.

### 2.6 Histological staining and immunohistochemical staining

H&E staining was used to evaluate histochemical changes. Safranin O-fast green staining and toluidine blue staining were performed to determine changes in proteoglycans distribution. After euthanizing the experimental mice using cervical dislocation, the ankle joints were immediately dissected and carefully cleaned of attached muscles and connective tissues. The joints were then fixed in 4% PFA for 24 hours and decalcified in 10% EDTA for 7 days. The tissues were dehydrated, embedded in paraffin, and sectioned into 5-µm thick slices. Subsequently, the sections were stained with H&E, Safranin O-fast green, or Toluidine blue staining dyes. Finally, images from each group were captured using a microscope.

For immunohistochemical Staining, tissue sections from each group were first incubated with 3% hydrogen peroxide for 10 minutes to block endogenous peroxidase activity, followed by blocking with 5% BSA for 30 minutes. The sections were then incubated overnight at 4°C with monoclonal antibodies against MPO (1:100), NE (1:100). Afterward, HRP-conjugated secondary antibodies were applied for 1 hour. Visualization was performed using 3,3‐diaminobenzidine (DAB) or 3-amino-9-ethylcarbazole (AEC) chromogen (Solarbio, China). Images were captured using a microscope for each group.

### 2.7 Quantitative PCR

Total RNA was extracted from frozen tissue samples using TRIzol reagent (Invitrogen) according to the standard protocol. Briefly, tissues were rapidly ground in liquid nitrogen and homogenized in 1 mL of TRIzol reagent. After incubation for 5 minutes at room temperature, chloroform was added (200 μL per 1 mL TRIzol), and the mixture was vigorously shaken for 15 seconds, followed by another 5-minute incubation. The samples were centrifuged at 13,000 × g for 15 minutes at 4 °C. The aqueous phase was carefully transferred to a new tube, mixed with an equal volume of cold isopropanol, and incubated for 10 minutes at 4 °C. RNA was pelleted by centrifugation at 13,000 × g for 10 minutes at 4 °C. The pellet was washed once with 75% ethanol, air-dried for 3–5 minutes, and finally dissolved in DEPC-treated water. RNA concentration and purity were determined using a microspectrophotometer.

Total RNA was extracted from tissues and cells with Trizol reagent (cat#: 9109; Invitrogen, Carlsbad, CA, USA) and reverse-transcribed into cDNA using the PrimeScript™ RT Reagent Kit (cat#: RR037A; TAKARA BIO INC, Shiga, Japan) according to the manufacturer’s instructions. The primer sequences used in this study are listed in **Table 1**. The amplification protocol consisted of an initial step at 95°C for 10 min, followed by 40 cycles of denaturation at 95°Cfor 15 s and annealing/extension at 60°C for 1 min. Gene expression levels were calculated using the 2-ΔΔCt method, with GAPDH as the internal reference gene. All reactions were carried out with three technical replicates.

**Table 1:** Primer sequences for qRT-PCR.

Primer name	Sequenc (5’ to 3’)
MPO	F: CCACACCCTCATCCAACCCTTC
	R: CCACCTTCCAACACGACTCTCC
NE	F: GAGGAGGCTGTGGATCTGGATTG
	R: CTCTCGGTCTTTGGGATGGGTAAG
MMP3	F: ACGATGATGAACGATGGACAGAGG
	R: GCCTTGGCTGAGTGGTAGAGTC
MMP13	F: ATACTACCATCCTGCGACTCTTGC
	R: CGGAGCCTGTCAACTGTGGAG
β-Actin	F: TATGCTCTCCCTCACGCCATCC
	R: GTCACGCACGATTTCCCTCTCAG
IL-1β	F: CACCTCACAAGCAGAGCACAAG
	R: GCATTAGAAACAGTCCAGCCCATAC
IL-6	F: GAAACCGCTATGAAGTTCCTCTCTG
	R: GTATCCTCTGTGAAGTCTCCTCTCC

### 2.8 Western blotting

Protein extraction was performed from ankle joint tissues using ice-cold RIPA lysis buffer supplemented with PMSF (100:1). The tissues were homogenized mechanically at 10,000 rpm under ice-cold conditions at a tissue-to-buffer ratio of 1:5 (w/v), followed by incubation on ice for 30 minutes. The homogenate was centrifuged at 12,000 × g for 15 min at 4°C to collect the supernatant. Protein concentration was determined spectrophotometrically. Denaturation was carried out by adding 5× SDS loading buffer at a 4:1 ratio and heating at 100°C for 10 min. Samples were stored at -20°C until further use. Proteins were separated by SDS-PAGE using gels prepared with a commercial fast preparation kit and electrically transferred to NC membranes under wet conditions. After blocking with rapid blocking buffer for 15 min at room temperature, membranes were incubated with specific primary antibodies at 4°C overnight. Following washes with PBST, HRP-conjugated secondary antibodies were applied for 1 h at room temperature. Protein bands were visualized via ECL detection. Quantification was performed using ImageJ software, with β-Actin serving as the internal control for normalization.

### 2.9 *In vitro* assay

#### 2.9.1 *Ex vivo* NETosis assay using primary mouse neutrophils

Primary neutrophils were isolated from the bone marrow of healthy male DBA/1 mice (8–10 weeks old) using a commercial mouse bone marrow neutrophil isolation kit (Solarbio, Cat. No. P8550). Briefly, bone marrow cells were flushed from the femurs and tibiae with ice-cold PBS. After red blood cell lysis, the cell suspension was subjected to density gradient centrifugation at 700 × g for 30 minutes at 4 °C. The neutrophil-rich layer was collected, washed, and resuspended in RPMI-1640 medium supplemented with 1% fetal bovine serum (FBS) and 1% penicillin-streptomycin. Cell viability was >95% (as determined by trypan blue exclusion), and purity was >90% (as assessed by Wright-Giemsa staining and flow cytometry for Ly6G). To induce NETosis, cells were seeded at a density of 2×10^6^ per well on poly-L-lysine-coated coverslips. After cell attachment, the medium was replaced with serum-free RPMI-1640. Subsequently, cells were pretreated with catalpol (10, 20, or 40 μM) or vehicle (DMSO, <0.1%) for 1 hour, followed by stimulation with 100 nM phorbol 12-myristate 13-acetate (PMA) for 4 hours (28) Control groups included untreated cells and cells treated with catalpol or vehicle alone; in some experiments, the PAD4 inhibitor Cl-Amidine (200 μM) was used. After stimulation, cells were fixed with 4% paraformaldehyde. Extracellular traps (ETs) were visualized by immunofluorescence staining using primary antibodies against citrullinated histone H3 (Cit-H3; 1:500) and myeloperoxidase (MPO; 1:200), followed by fluorescent secondary antibodies and DAPI counterstaining. Images were acquired using a confocal microscope. ETs were identified as extracellular fibrous structures where DAPI-stained DNA colocalized with Cit-H3 and MPO.

### 2.10 Chondrocyte culture, NETs-conditioned medium preparation, and treatment

ATDC5 chondrocytes were maintained in DMEM supplemented with 10% FBS and 1% penicillin-streptomycin at 37 °C under 5% CO_2_. NETs-conditioned medium (NETs-CM) was generated by stimulating primary mouse neutrophils (2 × 10^6^ cells/mL) with 100 nM PMA in serum-free RPMI-1640 for 4 h. The supernatant was collected, sequentially centrifuged (300 × g for 5 min, then 2000 × g for 10 min) to remove cells and debris, and filtered through a 3 kDa cut-off filter to eliminate residual PMA. Control conditioned medium (Ctrl-CM) was prepared from unstimulated neutrophils following the same procedure. For treatment, ATDC5 cells were serum-starved for 12 h upon reaching 70–80% confluence and then incubated with NETs-CM or Ctrl-CM (diluted 1:1 with serum-free DMEM/F-12) in the presence or absence of 10 μM catalpol for 24 h, The concentration of catalpol was selected based on our previous study demonstrating its efficacy in protecting chondrocytes against LPS-induced injury (29). Following treatment, intracellular ROS levels were measured using the DCFH-DA probe (10 μM, 30 min) with fluorescence quantified at Ex/Em = 488/525 nm. Mitochondrial membrane potential (ΔΨm) and apoptosis were assessed simultaneously using a commercial kit (Beyotime, Cat# C1071M) according to the manufacturer’s instructions, involving staining with MitoTracker Red CMXRos, Annexin V-FITC, and Hoechst 33342. In addition, protein expression of BAX, BCL2, Cleaved-Caspase-3, and MMP3 was analyzed by Western blot as described in the general methods.

### 2.11 Statistical analysis

Data management and analysis were performed using SPSS software. The data are expressed as the mean ± standard deviation (SD). Graphs were drawn using GraphPad Prism. Every experiment was repeated at least three times. All data were first tested for normality using the Shapiro-Wilk test. Data conforming to a normal distribution with homogeneity of variances were analyzed using the Student’s t-test (for comparisons between two groups) or one-way analysis of variance (ANOVA) followed by Tukey’s *post hoc* test (for comparisons among multiple groups). Data not conforming to a normal distribution were analyzed using the Mann-Whitney U test or the Kruskal-Wallis test. The specific test used is indicated in the legend of each figure.

A p-value of 0.05 or less was considered statistically significant.

## 3 Results

### 3.1 Effects of catalpol on ankle swelling and bone damage in collagen-induced arthritic mice

**Figure 1:**
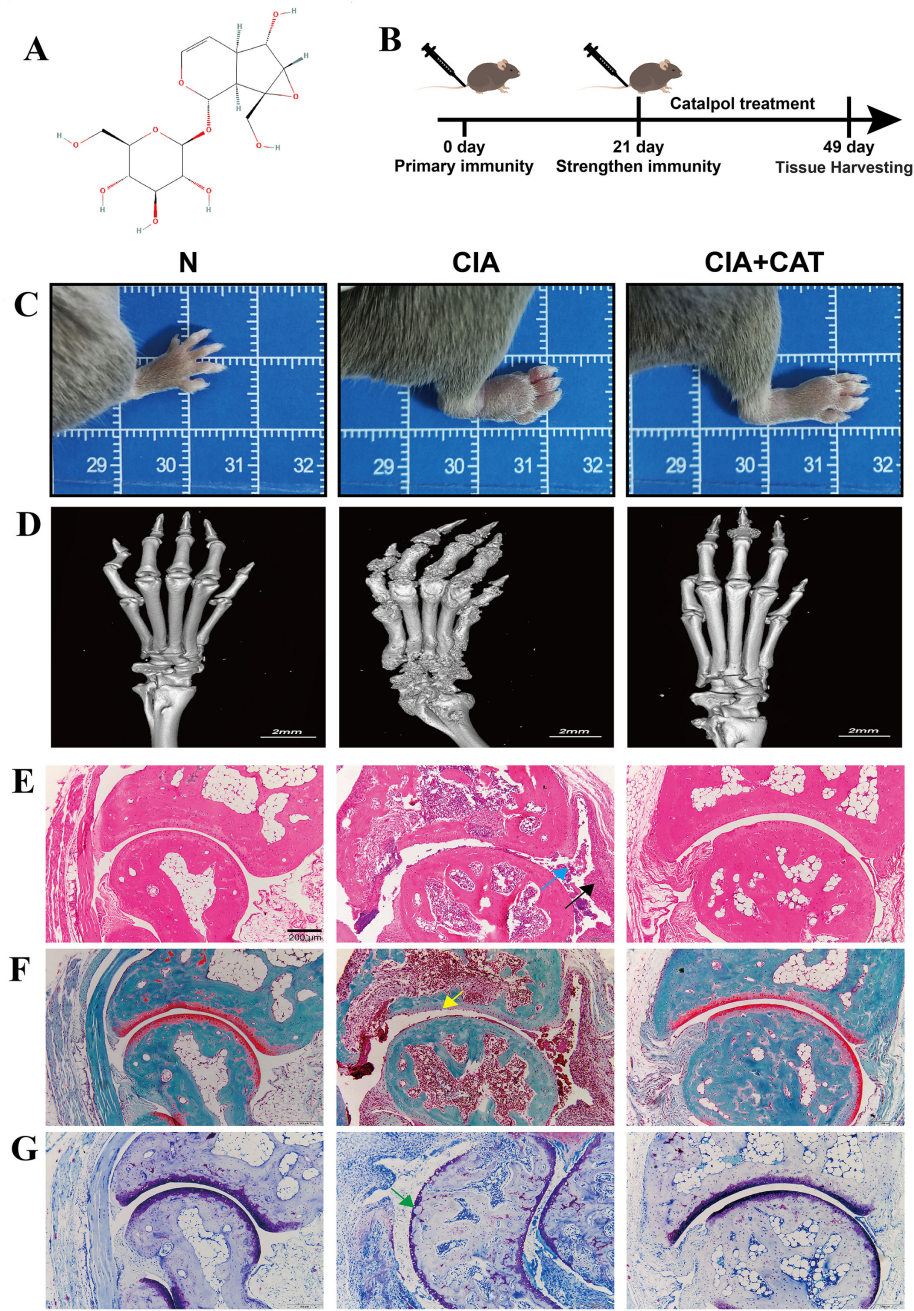
Effects of catalpol on ankle swelling and bone damage in collagen-induced arthritic mice. **(A)** the chemical structure of catalpol **(B)** the process of the experimental scheme **(C)** Representative photographs of hind paws from CIA mice. **(D)** Three-dimensional micro-CT reconstruction images comparing structural changes in the ankle joints among different groups, including bone surface morphology, cortical bone continuity, and bone erosion. **(E)** HE staining revealed synovial pathological changes. The normal group (N) exhibited intact synovial architecture. The CIA group demonstrated synovial hyperplasia (black arrows), inflammatory cell infiltration (blue arrows), and pannus formation. These pathological alterations were markedly ameliorated in the CAT group. **(F)** Safranin O-fast green staining demonstrated GAGs distribution. Reduced staining intensity (yellow arrows) in the CIA group indicated GAGs depletion, whereas the CAT group showed enhanced and homogeneous staining, suggesting improved matrix synthesis. **(G)** Toluidine blue staining evaluated proteoglycan content. The CIA group displayed superficial cartilage erosion with faint staining (green arrows) and disorganized chondrocyte arrangement. In contrast, the CAT group exhibited intensified staining along with improved cartilage thickness and structural integrity. (Scale bar: 200 μm).

After booster immunization on day 21, the mice in the model group exhibited severe arthritis symptoms. Initially (one week after immunization), the symptoms were characterized by redness and swelling of the hind paw, which progressively worsened. By two weeks post-immunization, the inflammation peaked, spreading to the entire hind limb and front paw. In severe cases, the mice experienced mobility impairment, and some were unable to stand (**Figure 1A**). Micro-CT three-dimensional reconstruction further revealed that the ankle joints of mice in the model group showed typical bone destruction features, including rough bone surfaces, cortical bone fractures, and local bone erosion. In contrast, the control group mice had intact bone structures and normal joint spaces (**Figure 1B**). Notably, after treatment with CAT (30 mg/kg), the mice not only showed a significant reduction in joint swelling, but Micro-CT analysis also demonstrated marked improvement in bone damage, characterized by smoother bone surfaces and reduced bone erosion.

### 3.2 Therapeutic effects of CAT on ankle joint tissues in CIA mice

To assess the therapeutic effect of CAT on the ankle joint tissue of CIA mice, HE staining, Fast Green Safranin O staining, and Toluidine Blue staining were used to observe the ankle joint tissue of CIA mice. As shown in **Figures 1C, D**, both gross photographs and CT imaging of the mouse hind limbs revealed significant RA symptoms in the model group. In contrast, the treatment groups exhibited markedly reduced signs of disease, demonstrating the potent protective effects of the compound. The HE staining results showed that, compared with the normal group (N), the CIA group exhibited significant pathological changes in the synovial membrane, including extensive infiltration of inflammatory cells (blue arrow), hyperplasia of synovial cells, and tissue thickening (black arrow). The proliferated synovium formed an invasive pannus structure. However, in the CAT-treated group, the above-mentioned inflammatory indicators were significantly improved (**Figure 1E**). Combined analysis with Fast Green Safranin O and Toluidine Blue staining revealed the protective effect of CAT on the cartilage in the ankle joint of CIA mice. Fast Green Safranin O staining showed that the distribution of sulfated glycosaminoglycans in the cartilage matrix was significantly reduced in the CIA group (yellow arrow indicating weakened staining intensity). In contrast, the CAT group showed increased staining intensity with a more uniform distribution, suggesting that CAT might promote the synthesis of sulfated glycosaminoglycans or inhibit their degradation, while also improving cartilage surface irregularities (**Figures 1F, G**). Toluidine Blue staining further confirmed that the CIA group exhibited pathological features such as proteoglycan loss (green arrow indicating faint staining), rough cartilage surface, reduced thickness, and disorganized cell arrangement, while the CAT treatment group showed deep and uniform staining, improved surface structure, regular cell arrangement, and increased cartilage thickness. The results of both staining methods consistently demonstrated that CAT effectively maintains the homeostasis of cartilage matrix components (sulfated glycosaminoglycans and proteoglycans), protects the integrity of cartilage tissue structure, and alleviates joint damage caused by CIA (**Figure 1G**).

### 3.3 Effect of CAT on the formation of NETs in the ankle joints of CIA mice

**Figure 2:**
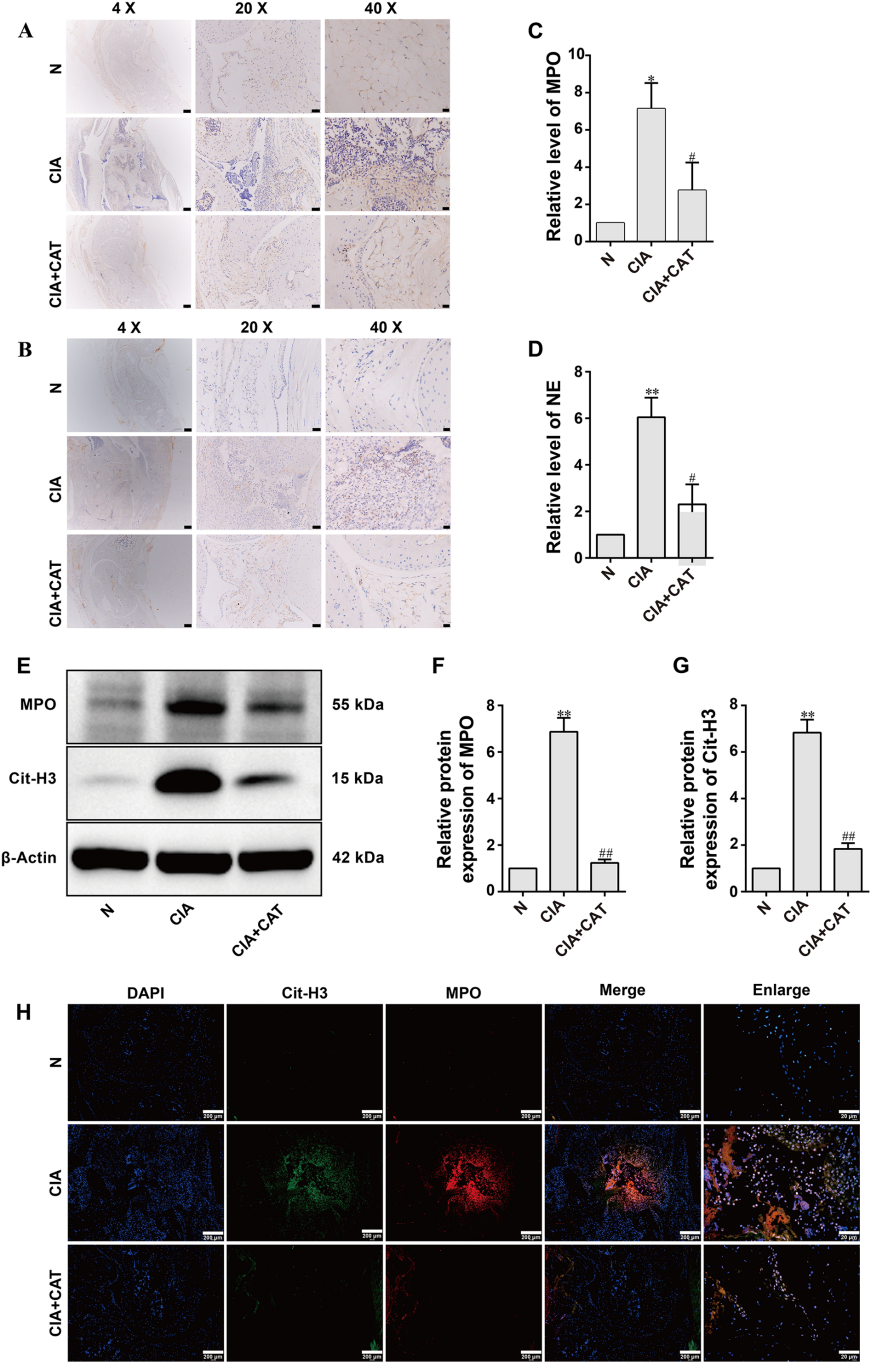
Effects of catalpol on NETs formation in the ankle joints of CIA mice. **(A, B)** Immunohistochemical staining of MPO and NE. **(C, D)** Statistical analysis of MPO and NE mRNA levels. **(E)** Representative Western blot images. **(F, G)** Statistical analysis of MPO and Cit-H3 protein levels by Western blot. Data are presented as mean ± SD from three independent experiments (n=3 per group). Statistical analysis was performed using Kruskal-Wallis test followed by Dunn’s *post hoc* test (applied due to the limited sample size).*P < 0.05, **P < 0.01 vs. N group; ^#^*P* < 0.05, ^##^*P* < 0.01 vs. CIA group (n=6 per group). **(H)** Representative immunofluorescence images of NETs in the ankle joints of CIA mice. DAPI (blue), Cit-H3 (green), and MPO (red). Scale bars: 200 μm and 20 μm.

Immunohistochemical analysis revealed a marked increase in the expression of myeloperoxidase (MPO) and neutrophil elastase (NE) within both the synovial layer and joint cavity of the ankle in the CIA model group compared to the normal controls (**Figures 2A, B**). Consistent with these findings, Western blot and qRT-PCR results indicated that the protein and transcript levels of citrullinated histone H3 (Cit-H3), MPO, and NE were significantly upregulated in CIA mice (**Figures 2C–G**). Catalpol (CAT) treatment effectively attenuated the increase in these markers. Furthermore, immunofluorescence double staining confirmed prominent co-localization of Cit-H3 and MPO in the inflammatory areas of CIA mice (**2H**), an effect that was substantially reduced following CAT administration, as evidenced by diminished fluorescence intensity and co-localization signals.

### 3.4 Effects of catalpol on inflammatory factors in ankle joint tissues of CIA mice

**Figure 3:**
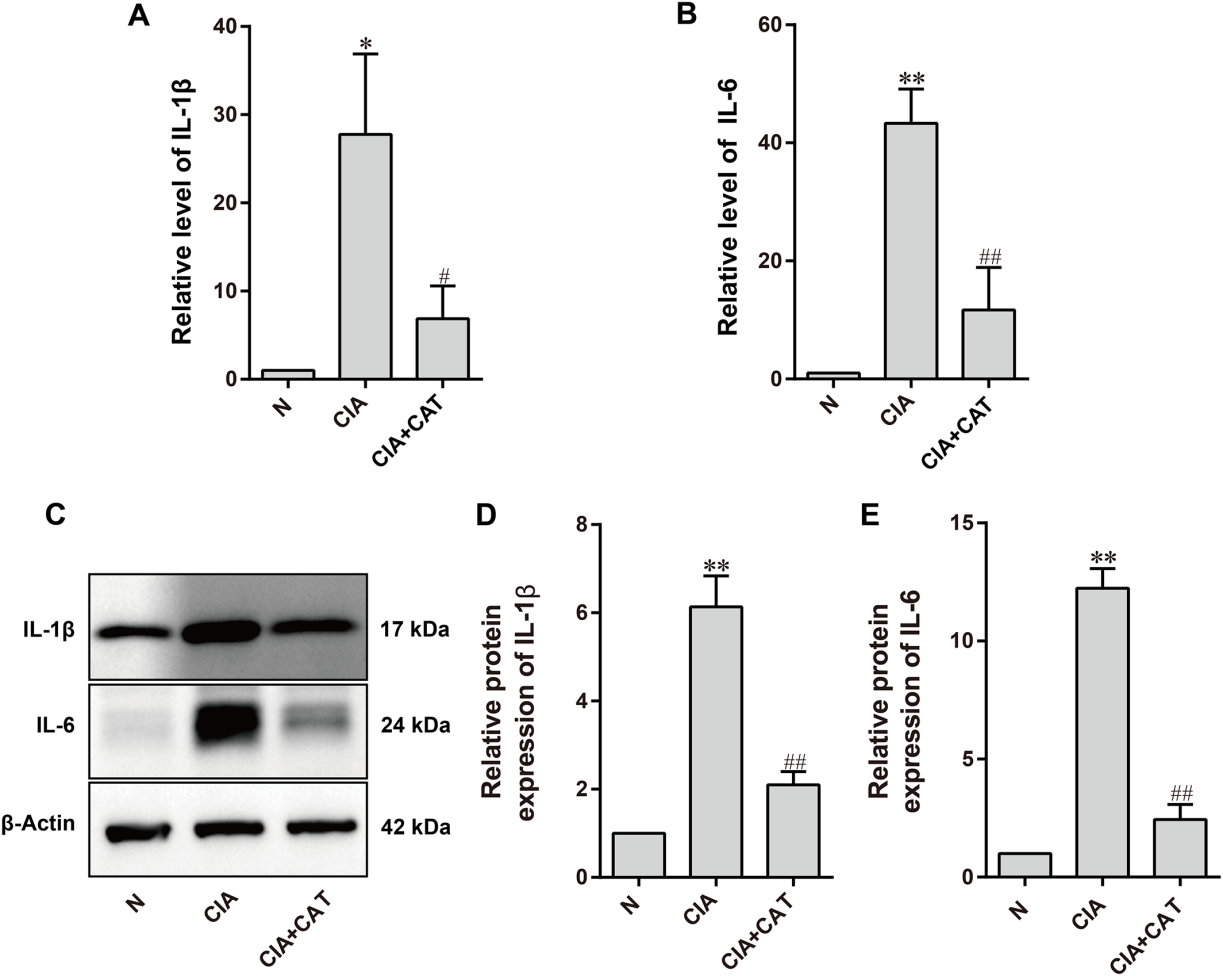
Effects of catalpol on inflammatory cytokine release and inflammatory proteins in CIA mice. **(A, B)** Statistical analysis of mRNA levels of IL-1β and IL-6. **(C)** Representative Western blot images. **(D, E)** Statistical analysis of protein expression levels of IL-1β and IL-6. Data (mean ± SD, n=6 mice/group) were analyzed by one-way ANOVA followed by Tukey’s *post hoc* test. *P < 0.05, **P < 0.01; compared with the CIA group, ^#^*P* < 0.05, ^##^*P* < 0.01 (n=6 per group).

RA is a systemic autoimmune disease, with chronic synovitis, production of autoantibodies, and the release of pro-inflammatory cytokines being key pathological features. These factors collectively contribute to the destruction of articular cartilage and surrounding bone (30). Synovial fibroblasts, through direct or indirect mechanisms, produce large amounts of pro-inflammatory cytokines such as TNF-α, IL-1β, IL-17, and IL-6, which are involved in the local and systemic inflammatory responses of RA (31). In turn, these pro-inflammatory cytokines stimulate abnormal synovial proliferation, upregulate matrix metalloproteinases and adhesion molecule expression, exacerbate inflammation, and promote synovial hyperplasia, cartilage degradation, and bone erosion (32, 33). qRT-PCR analysis revealed that the levels of IL-1β and IL-6 in the ankle joint tissue of CIA mice were significantly elevated compared to the normal group. In contrast, the CIA+CAT group showed a significant reduction in IL-1β and IL-6 levels compared to the CIA group (**Figures 3A, B**). Western blot results further indicated that the expression of IL-1β and IL-6 proteins in the CIA group was significantly upregulated compared to the normal group, whereas CAT intervention led to a marked downregulation of these pro-inflammatory proteins (**Figures 3C–E**).

### 3.5 Catalpol alleviates joint injury in CIA mice by synergistically inhibiting cartilage degradation and bone resorption

**Figure 4:**
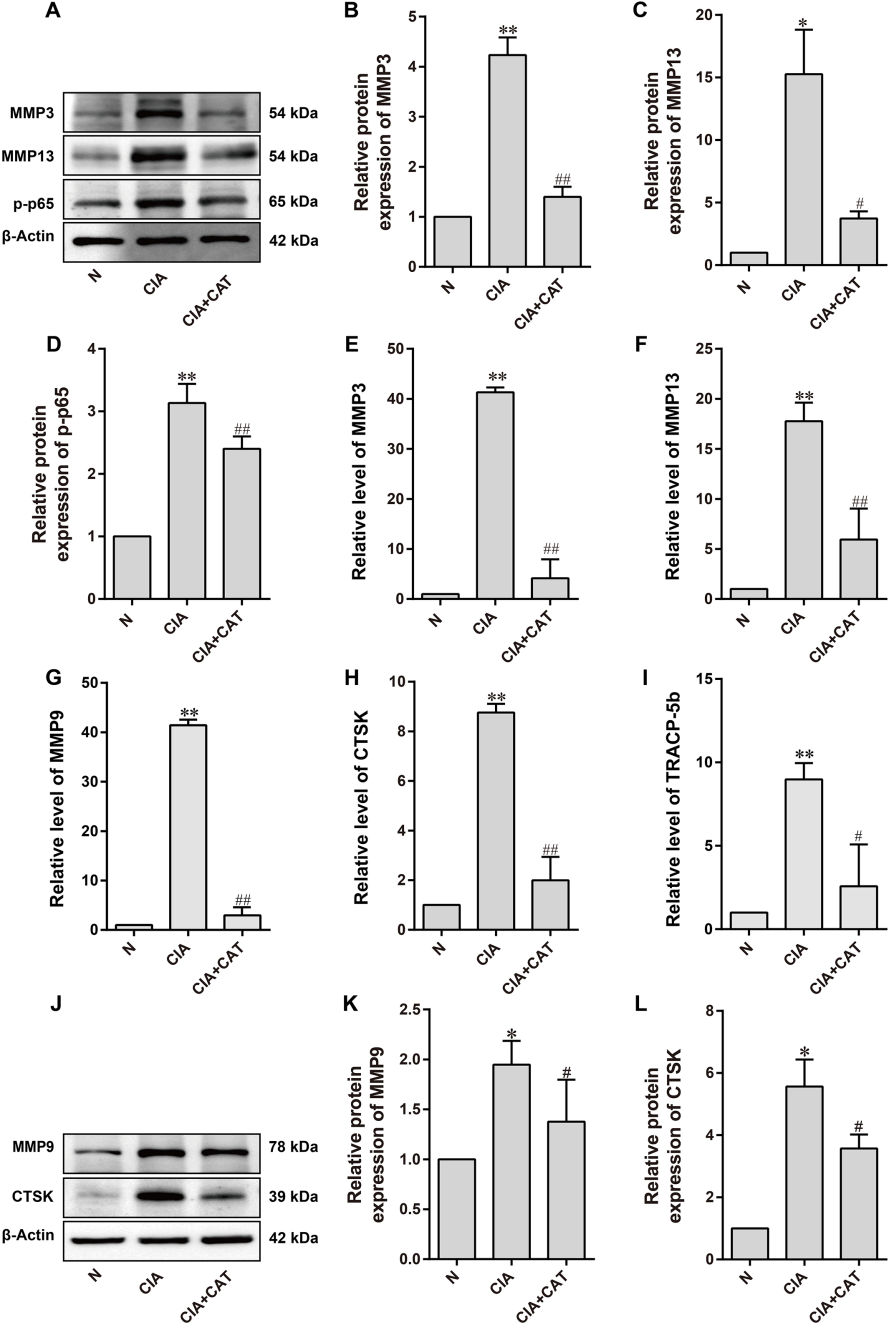
Effects of catalpol on the expression levels of cartilage-related and osteoclast-related factors in CIA mice. **(A)** Representative Western blot images of cartilage-related proteins. **(B–D)** Statistical analysis of MMP3, MMP13, and p-p65 protein expression levels. **(E, F)** Statistical analysis of MMP3 and MMP13 mRNA levels in CIA mice. **(G–I)** Statistical analysis of MMP9, CTSK, and Tracp-5b mRNA levels in CIA mice. **(J)** Representative Western blot images of osteoclast-related proteins. **(K, L)** Statistical analysis of MMP9 and CTSK protein expression levels. Data (mean ± SD, n=6 mice/group) were analyzed by one-way ANOVA followed by Tukey’s *post hoc* test. *P < 0.05, **P < 0.01 vs. N group; ^#^*P* < 0.05, ^##^*P* < 0.01 vs. CIA group (n=6 per group).

In the pathological progression of RA, cartilage degradation and bone erosion are interrelated key events, with matrix metalloproteinases (MMPs) and osteoclast activity-related molecules (such as CTSK and Tracp-5b) driving this destructive process. MMP3 and MMP13 play central roles in cartilage destruction. MMP3 degrades extracellular matrix (ECM) components such as proteoglycans and fibronectin, and activates other MMPs, promoting the invasiveness of synovial fibroblasts and exacerbating cartilage matrix degradation (34), MMP13 specifically cleaves type II collagen, directly destroying cartilage structure while also promoting the degradation of proteoglycans, further accelerating cartilage degeneration (35). Furthermore, activation of the NF-κB signaling pathway triggered by inflammatory cytokines (such as IL-1β and TNF-α) enhances the expression of MMP3 and MMP13, creating a positive feedback loop that exacerbates cartilage damage (36). At the same time, osteoclast-mediated bone erosion is closely linked to cartilage degradation. MMP9, an important member of the MMP family, not only participates in the degradation of cartilage matrix but also promotes osteoclast migration and bone resorption (37). CTSK, a protease secreted specifically by osteoclasts, directly degrades collagen in the bone matrix, while Tracp-5b serves as a marker of osteoclast activity, reflecting the intensity of bone resorption (38, 39). In RA, persistent synovial inflammation promotes osteoclast differentiation and activation, leading to bone erosion, while inflammatory mediators released from degraded cartilage further activate osteoclasts, forming a vicious cycle of “cartilage destruction - bone erosion.” The results indicate that CAT intervention significantly reduced the expression of MMP3, MMP13, MMP9, CTSK, and Tracp-5b in the CIA mouse model, while inhibiting the NF-κB signaling pathway (**Figures 4A–L**). This suggests that CAT may alleviate joint destruction in RA through dual mechanisms: (1) inhibiting MMP3/MMP13-mediated ECM degradation to reduce cartilage damage, and (2) decreasing MMP9, CTSK, and Tracp-5b expression to suppress osteoclast-mediated bone resorption. Given the reciprocal promotion of cartilage and bone destruction in RA, CAT’s multitarget action may effectively delay the progressive damage to joint structures by disrupting the “inflammation-cartilage degradation-bone erosion” feedback loop.

### 3.6 Catalpol inhibits PMA-induced NETs formation in neutrophils

**Figure 5:**
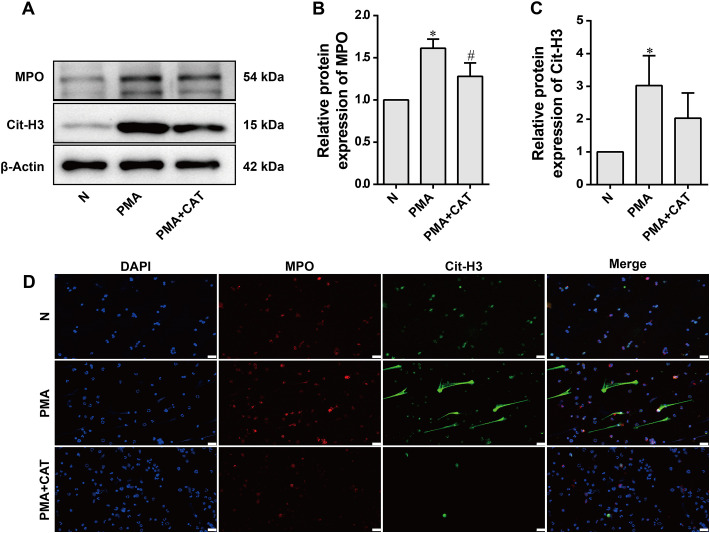
Effect of CAT on NETs-associated marker proteins. **(A)** Representative Western blot images. **(B, C)** Statistical analysis of MPO and Cit-H3 protein expression. Data are presented as mean ± SD from three independent experiments (n=3 per group). Statistical analysis was performed using Kruskal-Wallis test followed by Dunn’s *post hoc* test (applied due to the limited sample size). *P < 0.05 vs. N group; ^#^*P* < 0.05 vs. PMA group (n=3 per group). **(D)** Immunofluorescence co-localization staining of Cit-H3 and MPO. Representative images showing Cit-H3 (green), MPO (red), and DAPI (blue) staining. Scale bar: 20 μm.

To investigate whether CAT inhibits NET formation in neutrophils stimulated by PMA, we assessed the expression levels of key NET markers, Cit-H3 and MPO, using Western blot and immunofluorescence assays. Western blot results (**Figures 5A–C**) showed that, after PMA induction (28), the expression levels of Cit-H3 and MPO were significantly increased. In the PMA+CAT group, the expression of Cit-H3 showed a downward trend, and MPO expression was significantly reduced. To further validate the inhibitory effect of CAT on NET formation, immunofluorescence staining was performed to assess the co-localization of Cit-H3 and MPO. As shown in **Figure 5D** , after 4 hours of stimulation with 100 nM PMA, Cit-H3 (green) and MPO (red) exhibited significant co-localization signals, forming typical NET structures. However, in the PMA+CAT group, the co-localization signals of Cit-H3 and MPO were markedly diminished, and the formation of NET structures was significantly reduced. These results further confirm that CAT plays a crucial role in inhibiting NET formation in the context of anti-RA therapy.

### 3.7 Mechanistic insights into NETs-mediated chondrocyte damage in RA and the protective role of CAT

**Figure 6:**
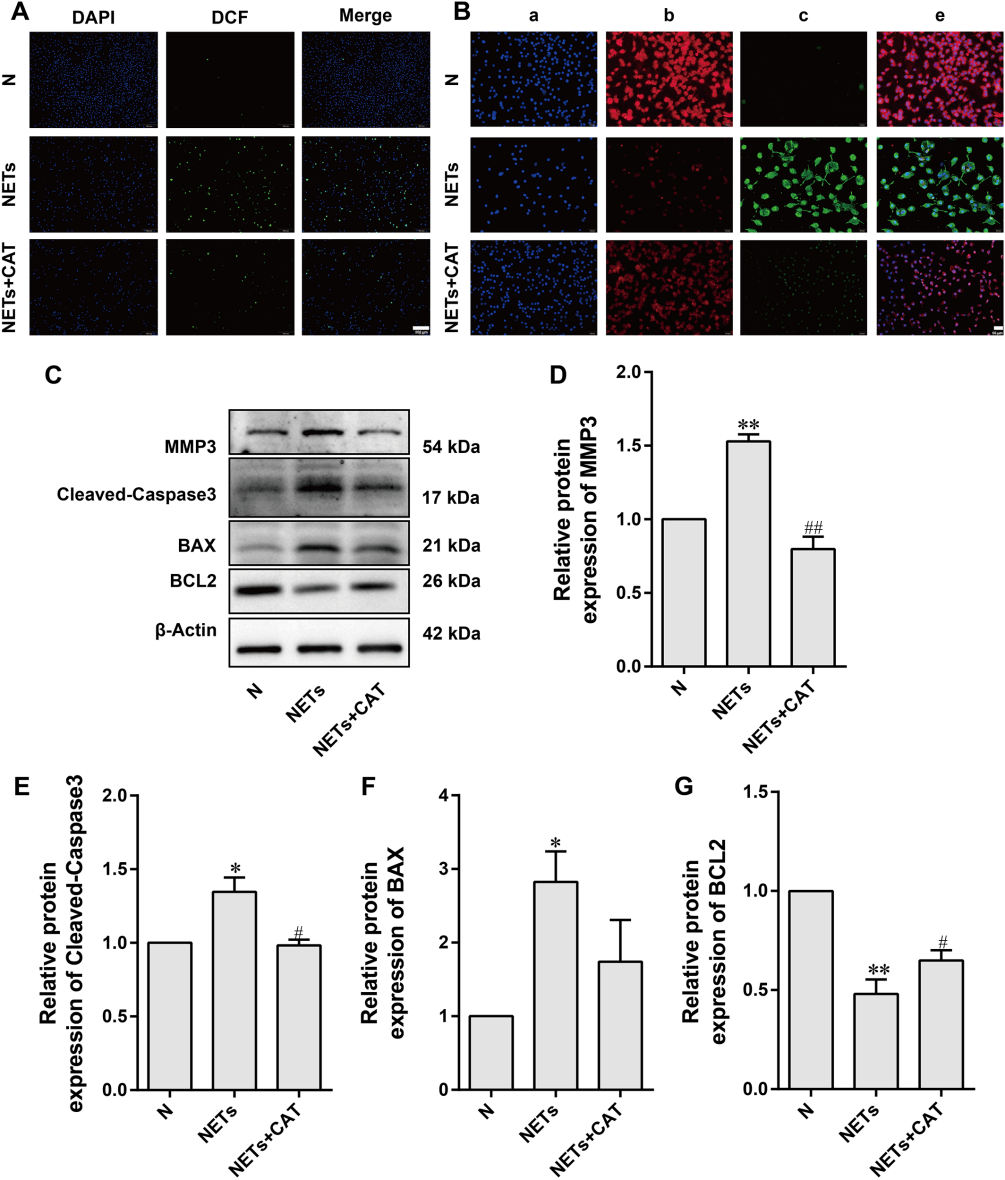
Mechanistic insights into NETs-mediated chondrocyte damage in RA and the protective role of CAT. **(A)** Representative images of DCFH-DA staining in ATDC5 cells showing DAPI (blue) and DCF (green). Scale bar: 200 μm. **(B)** Fluorescence images of mitochondrial membrane potential and apoptosis in ATDC5 cells: **(a)** Nuclear staining (Hoechst 33342), **(b)** Mitochondrial membrane potential staining (Mitotracker-Red), **(c)** Phosphatidylserine exposure staining (Annexin V-FITC), **(d)** Merged image of Hoechst 33342, Mitotracker-Red and Annexin V-FITC staining. Scale bar: 50 μm. **(C)** Representative Western blot bands. **(D–G)** Quantitative analysis of MMP3, Cleaved-Caspase3, BAX and BCL2 protein expression levels. Data (mean ± SD, n=6 mice/group) were analyzed by one-way ANOVA followed by Tukey’s *post hoc* test. *P < 0.05, **P < 0.01 vs. N group; ^#^*P* < 0.05, ^##^*P* < 0.01 vs. NETs group.

To elucidate the mechanism by which NETs participate in cartilage damage through oxidative stress and apoptosis pathways, this study cultured chondrocytes with PMA-induced NETs supernatant and systematically observed its biological effects. The DCFH-DA fluorescent probe assay revealed a significant increase in green fluorescence intensity in chondrocytes treated with NETs (**Figure 6A**), confirming that NETs can induce a burst of ROS. Based on previous studies identifying 10 μM CAT as an effective concentration to alleviate LPS-induced cartilage damage (29), this experiment demonstrated that this concentration of CAT significantly inhibited ROS production induced by NETs.

Further investigations showed that NETs led to a decrease in mitochondrial membrane potential (reduced Mitotracker-Red red fluorescence) and an increase in cell apoptosis (enhanced Annexin V-FITC green fluorescence), which CAT treatment was able to reverse (**Figure 6B**). Western blot analysis (**Figures 6C–G**) revealed that NETs significantly upregulated the pro-apoptotic proteins BAX and Cleaved-Caspase-3, while downregulating the anti-apoptotic protein BCL2. Notably, NETs also significantly increased the expression of matrix-degrading enzyme MMP3. After CAT intervention, the BAX/BCL2 ratio decreased, Cleaved-Caspase-3 expression was suppressed, and MMP3 expression was significantly reduced, indicating that CAT protects cartilage by simultaneously modulating both apoptotic and matrix degradation pathways.

### 3.8 Identification of key targets for CAT-mediated inhibition of NETs formation

**Figure 7:**
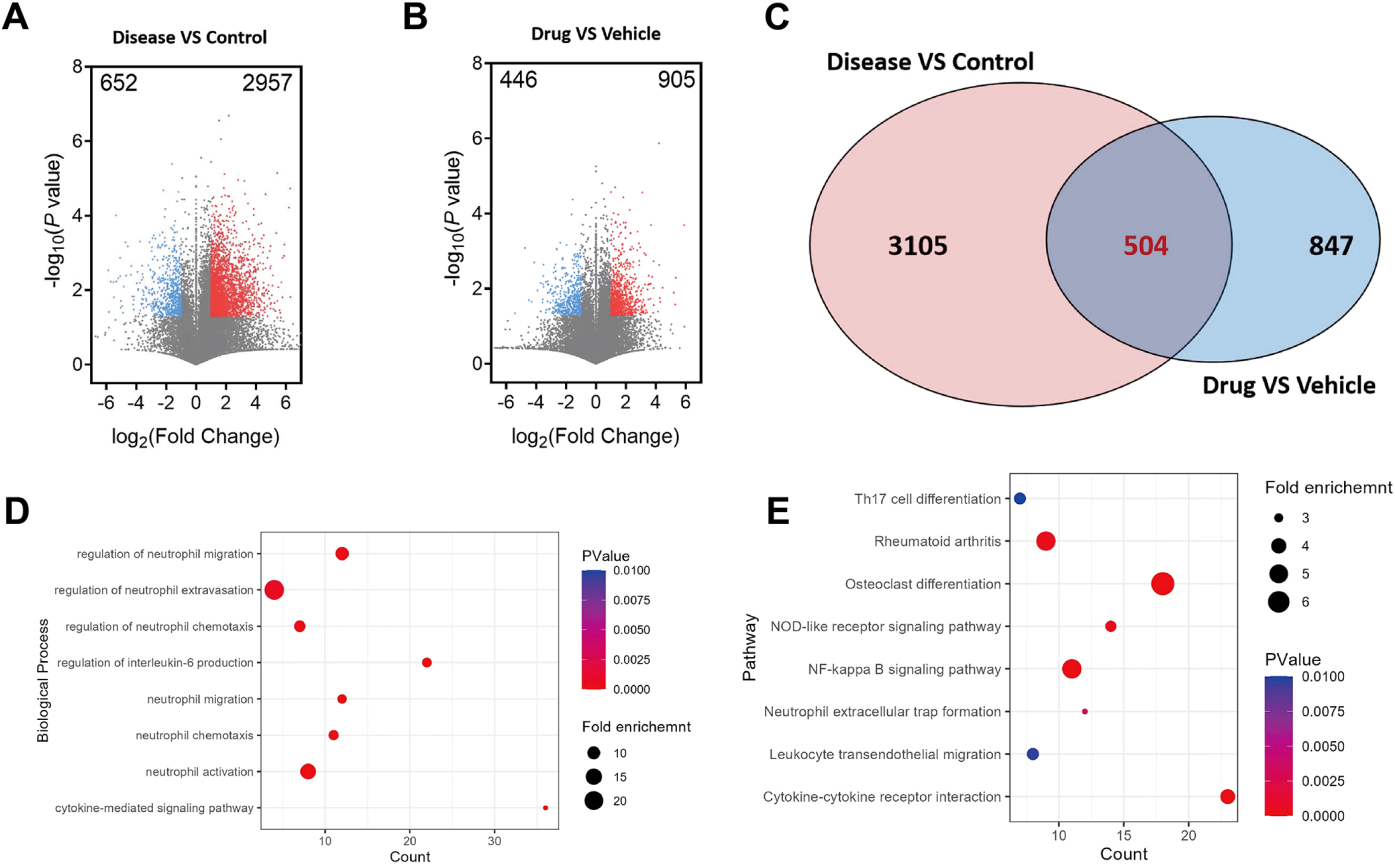
Identification of key targets for CAT-mediated inhibition of NETs formation. **(A)** Volcano plot comparing the normal and model groups. **(B)** Volcano plot comparing the model and CAT-treated groups. Each dot represents a gene, with red and blue dots indicating significantly upregulated and downregulated genes, respectively. The x-axis shows the log2 fold change in gene expression, while the y-axis represents the statistical significance of differential expression (-log10 P-value). **(C)** Venn diagram illustrating overlapping differentially expressed genes. **(D, E)** Bubble plots of GO and KEGG enrichment analyses for differentially expressed genes. Bubble size corresponds to the number of genes, and color intensity reflects enrichment significance (-log10 P-value).

To explore the targets through which CAT inhibits NET formation, we conducted transcriptomic analysis on the ankle joint tissue of CIA mice. Gene expression data were presented as scatter plots, with significantly upregulated and downregulated genes highlighted in red and blue, respectively (**Figures 7A, B**). Compared to the normal group, the CIA group showed 2957 significantly upregulated genes. In the treatment group, 446 genes were significantly downregulated compared to the CIA group. These differential genes may be closely related to the pathological process of RA, reflecting the regulatory effect of the drug on RA pathology. To further identify the targets of CAT in inhibiting NET formation, we intersected the differential genes between the normal and model groups, as well as the model and treatment groups, resulting in 504 common genes (**Figure 7C**). GO enrichment and KEGG pathway analysis of these differential genes revealed that they were significantly enriched in biological processes related to neutrophils and PMNs, including neutrophil regulation, neutrophil chemotaxis, neutrophil migration and activation, and cytokine production and mediation processes. These results suggest that the differential genes primarily participate in regulating neutrophil inflammation and immune modulation functions (**Figure 7D**).

KEGG pathway analysis showed that the differential genes were significantly enriched in pathways related to Th17 cell differentiation, rheumatoid arthritis, osteoclast differentiation, NOD-like receptor signaling, NF-κB signaling, and NET formation (**Figure 7E**). These pathways are closely associated with inflammatory responses, immune regulation, and NET formation, further indicating the critical role of NETs in rheumatoid arthritis.

### 3.9 Catalpol suppresses PAD4 protein expression and reduces NETs release

PAD4 catalyzes histone citrullination, which promotes chromatin decondensation and cell membrane rupture, ultimately leading neutrophils to release NETs (neutrophil extracellular traps) (40, 41). To determine whether catalpol (CAT) reduces NET release by inhibiting PAD4 expression, we performed Western blot experiments with the following groups: normal group (N), PMA stimulation group (PMA), PMA+CAT group, PMA+Cl-Amidine group, and PMA+CAT+Cl-Amidine group. Cl-Amidine, a specific PAD4 inhibitor, irreversibly binds to the PAD4 active site, effectively inhibiting its enzymatic activity. Due to its high specificity for PAD4, Cl-Amidine has been widely used in NET formation studies (42, 43). In this experiment, we used a Cl-Amidine concentration of 200 μM, a dose that has previously been shown to significantly inhibit PAD4 activity (44).

**Figure 8:**
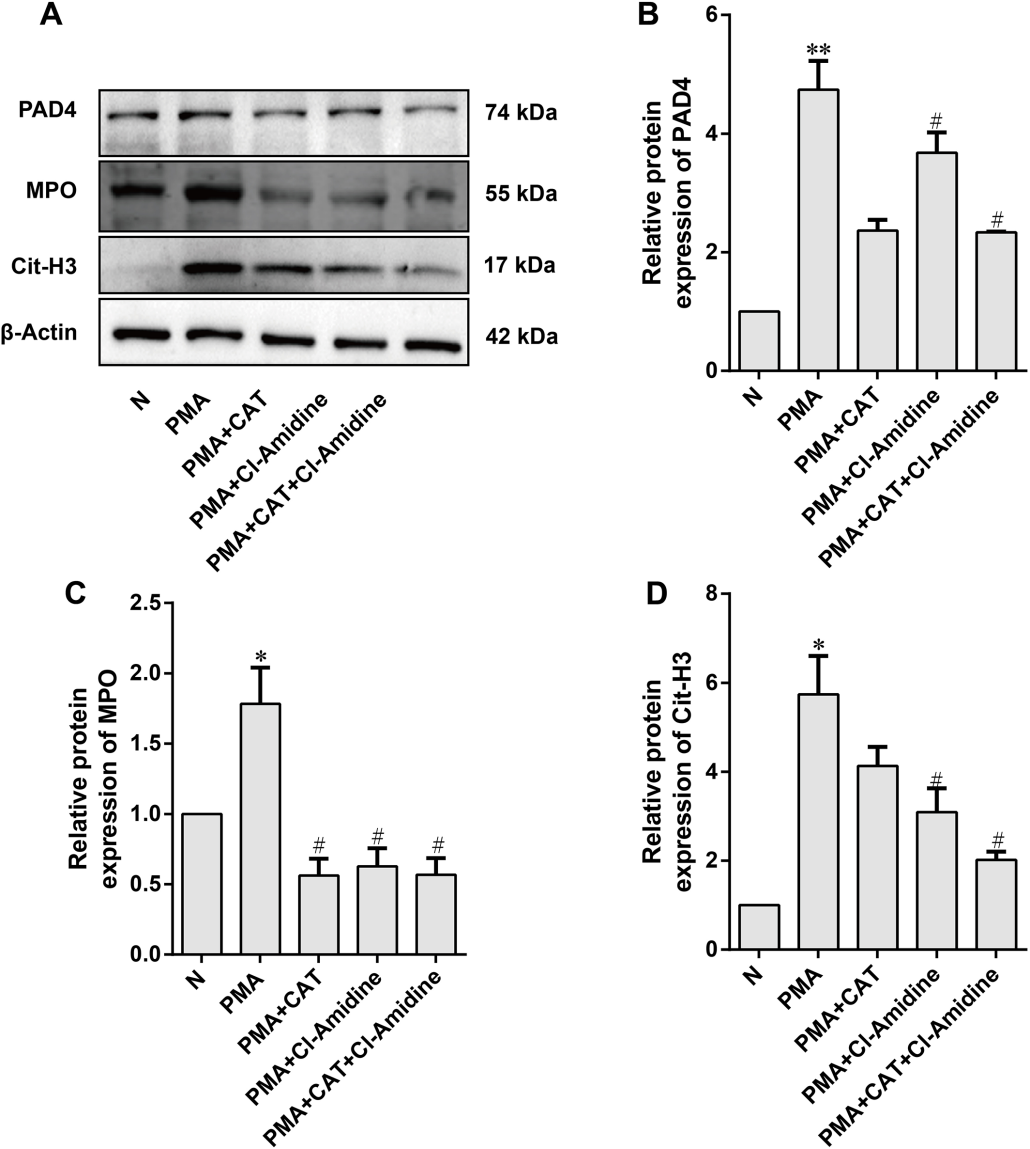
Effect of catalpol on PAD4 protein expression. **(A)** Representative Western blot images. **(B–D)** Statistical analysis of PAD4, MPO, and Cit-H3 protein expression levels. Data are presented as mean ± SD from three independent experiments (n=3 per group). Statistical analysis was performed using Kruskal-Wallis test followed by Dunn’s *post hoc* test (applied due to the limited sample size). *P < 0.05, **P < 0.01 vs. normal group (N); ^#^*P* < 0.05 vs. PMA-treated group.

The results (**Figures 8A–D**) demonstrated that PMA induction significantly increased the expression levels of PAD4, MPO, and Cit-H3, indicating effective upregulation of PAD4 and NET release. However, CAT treatment reduced PAD4 and Cit-H3 expression, and significantly decreased MPO expression (P < 0.05), suggesting that CAT inhibited PAD4 expression and suppressed NET release. Furthermore, the PAD4-specific inhibitor Cl-Amidine also significantly reduced the levels of PAD4, Cit-H3, and MPO. In the PMA+CAT+Cl-Amidine group, the combined treatment of CAT and Cl-Amidine further reduced the expression of PAD4, Cit-H3, and MPO, indicating that CAT may enhance the inhibition of NET formation by synergistically suppressing PAD4 expression. These findings suggest that CAT significantly reduces NET release by inhibiting PAD4 protein levels, providing experimental evidence for its role in suppressing NET formation.

## 4 Discussion

RA is a chronic autoimmune disorder primarily characterized by persistent joint inflammation, often presenting as symmetrical arthritis. It predominantly affects smaller joints, such as those in the hands, wrists, and knees, and is commonly associated with symptoms like morning stiffness, joint swelling, and pain. Without effective management, RA can lead to irreversible joint damage, deformities, functional impairment, and systemic complications, all of which significantly reduce the patient’s quality of life. The condition affects approximately 1 in 200 adults globally and is more prevalent in women, with a prevalence rate 2 to 3 times higher than in men. RA can develop at any age, though it most commonly manifests between the ages of 50 and 59. In addition to joint involvement, the disease may also impact various other organs, including the lungs, heart, blood vessels, skin, and eyes (45). Traditional treatments for rheumatoid arthritis rely on NSAIDs and corticosteroids, which can alleviate symptoms but have limited effects in slowing disease progression and promoting joint repair. In recent years, biologic agents and targeted therapies have significantly improved treatment outcomes. However, long-term use of NSAIDs and corticosteroids may lead to gastrointestinal issues and osteoporosis. Biologic agents are costly and carry a risk of increased infections and allergic reactions, while immunosuppressants are often associated with gastrointestinal discomfort and potential liver and kidney damage (5). As such, there is a need to explore novel drug targets for the treatment of RA.

Synovial inflammation is a key feature of RA, with various pro-inflammatory and immune-regulatory factors driving osteoclast formation and the degradation of bone and cartilage (46). Monocytes secrete high levels of pro-inflammatory cytokines, including TNF-α, IL-1β, and IL-6, and differentiate into inflammatory macrophages, exacerbating local synovial inflammation (47). IL-6, primarily produced by synovial fibroblasts, is closely associated with RA activity and promotes the release of additional pro-inflammatory mediators. It also stimulates B-cell differentiation into plasma cells, leading to the production of RA-specific autoantibodies (48). IL-17, secreted by Th17 cells, enhances IL-6 expression and induces synovial fibroblasts to release MMP1 and MMP3, accelerating ECM degradation in cartilage and bone. IL-17, in synergy with TNF-α and IL-1β, promotes osteoclastogenesis while inhibiting osteoblast function, disrupting the balance between bone formation and resorption, contributing to cartilage damage and bone erosion (49). Chronic inflammation in RA, driven by cytokines such as TNF-α, IL-1β, and IL-6, activates chondrocytes and synovial cells, enhancing MMP expression and ultimately degrading the cartilage matrix. Western blot analysis confirmed that CAT downregulated the expression of IL-1β, IL-6, MMP3, and MMP13 in joint tissue and inhibited the phosphorylation of the key NF-κB pathway protein p65. Notably, CAT also reduced the osteoclast-related markers CTSK and MMP9, indicating its multi-target intervention in bone metabolic imbalance.

Recent evidence increasingly highlights the significant role of the innate immune system, particularly neutrophils, in diseases such as RA (50). Neutrophil extracellular traps (NETs) are extracellular structures released by neutrophils in response to pathogens or other stimuli. Composed of DNA, histones, and various antimicrobial proteins, NETs are considered a key immune mediator in RA, contributing to the amplification of inflammation and autoimmune responses. MPO and NE, essential components of neutrophils, play antimicrobial roles in adaptive immunity by targeting pathogens (51, 52). When neutrophils are stimulated by pathogens, PMA, or lipopolysaccharides, arginine residues in the N-terminal tail of histone H3 undergo deimination, reducing the positive charge of the histone and disrupting its electrostatic interaction with the DNA phosphate backbone. This leads to chromatin decondensation and the release of MPO and NE into the extracellular space, forming NETs (53). Immunohistochemical staining of mouse ankle joints revealed enhanced MPO and NE staining in the synovium of CIA mice. Immunofluorescence staining showed significant co-localization of Cit-H3 and MPO in CIA synovial tissue and joint cavities, with signal intensity stronger than that in normal ankle joint tissue. CAT treatment reversed this phenomenon, suggesting that CAT not only alleviates joint inflammation but may also inhibit NET release by regulating Cit-H3 and MPO expression. Western blot analysis of mouse ankle joint samples further confirmed that the expression of Cit-H3 and MPO was significantly increased in CIA mice, but decreased following CAT treatment, providing additional evidence for CAT’s inhibitory effect on NETs. We assessed the dose-dependent inhibition of NETosis by catalpol via Western blot for Cit-H3. As shown in **Supplementary Figure 3**, Cit-H3 expression declined progressively with increasing CAT concentrations, with significant and maximal suppression at 10 µM. Although higher doses were not tested, the clear dose-response and significant effect at 10 µM support its selection as the optimal concentration for NETosis inhibition *in vitro*. Wu et al. reported that CAT at 20–80 µM maintained cell viability and suppressed nitrite release, selecting this range for mechanistic studies. Consistently, our data show that while NETosis was maximally inhibited at 10 µM, other functional parameters, such as proliferation, migration, invasion, and cytokine expression, required higher concentrations (20–80 µM) for full efficacy, suggesting that NET formation may be particularly sensitive to catalpol, possibly via direct modulation of NADPH oxidase or PAD4 signaling (24).

In addition, we investigated the role of NETs in cartilage damage in RA through mechanisms involving oxidative stress and apoptosis. Our findings suggest that NETs contribute significantly to the pathogenesis of cartilage damage by inducing oxidative stress and apoptosis in chondrocytes. The results indicate that NETs promote reactive oxygen species (ROS) production, which leads to mitochondrial dysfunction and apoptosis in chondrocytes. The elevated ROS levels observed in the NETs-treated chondrocytes, as shown by DCFH-DA staining, confirm that NETs are capable of inducing oxidative stress. In our study, mitochondrial dysfunction was further evidenced by the significant decrease in mitochondrial membrane potential, as shown by MitoTracker-Red staining, coupled with increased apoptotic signals from Annexin V-FITC staining. These findings align with the well-documented effects of oxidative stress on mitochondrial integrity and the initiation of apoptosis in various cell types. Interestingly, our data demonstrate that treatment with CAT effectively mitigated the detrimental effects of NETs. CAT treatment not only preserved mitochondrial membrane potential but also reduced the apoptotic signals induced by NETs. This suggests that CAT plays a protective role by scavenging ROS, thereby preventing mitochondrial dysfunction and apoptosis. These results highlight CAT as a potential therapeutic agent in mitigating the effects of NETs on chondrocyte viability and function. Western blot analysis further corroborated the involvement of apoptosis pathways in NETs-induced chondrocyte damage. We observed increased expression of the pro-apoptotic protein BAX and decreased expression of the anti-apoptotic protein BCL2 in the NETs-treated group. Additionally, the activation of caspase-3, indicated by the increased levels of Cleaved-Caspase-3, confirmed the induction of apoptosis. However, CAT treatment reversed these changes, as it restored BCL2 levels, reduced BAX expression, and inhibited Cleaved-Caspase-3 activation, providing additional evidence for the protective effect of CAT on chondrocytes. Furthermore, our findings show that NETs upregulate the expression of MMP3, suggesting that NETs may exacerbate cartilage matrix degradation. This is in line with previous studies indicating that NETs promote tissue degradation through the activation of MMPs. Importantly, CAT treatment attenuated the expression of MMP3, indicating that it not only protects against apoptosis but also prevents the degradation of cartilage matrix.

To investigate the target of CAT in inhibiting NETs formation, transcriptomic analysis was performed on ankle joint tissues from CIA mice. The results of GO enrichment analysis indicated that differentially expressed genes were significantly enriched in biological processes related to neutrophil polymorphonuclear (PMN) cells, including neutrophil regulation, neutrophil chemotaxis, and neutrophil migration. These findings suggest that the differentially expressed genes primarily participate in regulating the inflammatory response and immune modulation of neutrophils. KEGG pathway analysis further identified key pathways, including Th17 differentiation, NF-κB signaling, and NETs formation. These pathways are closely associated with inflammation, immune regulation, and the formation of NETs, further emphasizing the critical role of NETs in RA. Investigation of genes enriched in the NETs pathway revealed that PAD4 (peptidyl arginine deiminase 4) catalyzes histone citrullination, promoting chromatin decondensation and cell membrane rupture, thereby driving the release of NETs by neutrophils (40, 41). Cl-Amidine, a specific PAD4 inhibitor, was used in this study. Neutrophils were stimulated with PMA (phorbol myristate acetate) to induce NETs formation, followed by treatment with CAT and Cl-Amidine. Western blot analysis was performed to evaluate the protein expression levels of PAD4, MPO (myeloperoxidase), and Cit-H3 (citrullinated histone H3) to determine whether CAT could inhibit PAD4 and, subsequently, NETs formation. The results showed that following PMA induction, PAD4 expression in neutrophils was significantly elevated, along with increased expression of MPO and Cit-H3, indicating that upregulation of PAD4 is associated with enhanced NETs formation. After CAT treatment, the expression of PAD4, MPO, and Cit-H3 was significantly downregulated. Notably, when the concentration of CAT was 1/20th of that of Cl-Amidine, it significantly inhibited PAD4 expression and NETs formation. Additionally, when CAT and Cl-Amidine were co-applied to PMA-induced neutrophils, PAD4 expression was also significantly reduced. These results suggest that CAT and Cl-Amidine can act synergistically to inhibit PAD4 expression, thereby suppressing the formation of NETs. Beyond directly inhibiting neutrophil NETosis, suppression of PAD4 may also exert therapeutic effects by modulating adaptive immunity. Recent evidence (54) suggests that inhibiting PAD4 can influence dendritic cell antigen presentation function and regulate T cell responses. This implies that Catalpol, via PAD4 inhibition, might not only directly mitigate NET-driven inflammation and tissue damage but also indirectly modulate dysregulated antigen-specific T cell responses in RA. **Supplementary Figure 2** shows elevated PAD4 and PAD2 levels in CIA mice, consistent with their known roles in RA. Catalpol treatment significantly reduced PAD4 and modestly lowered PAD2, suggesting its therapeutic effect primarily involves specific PAD4 downregulation, though PAD2 modulation may also contribute, reflecting the complexity of PAD biology in inflammation (55). Catalpol is established as a modulator of inflammatory networks, particularly the NF-κB pathway. Correspondingly, our data show that catalpol treatment reduced NF-κB p65 phosphorylation and decreased levels of downstream cytokines IL-1β and IL-6 in mouse joints. These cytokines drive synovitis by priming neutrophils for NETosis and activating cartilage-degrading and bone-resorbing cells. We thus propose that catalpol exerts its primary anti-inflammatory effect via NF-κB inhibition, establishing an inhibitory microenvironment that simultaneously disrupts several pathogenic cycles: it attenuates cytokine-driven neutrophil recruitment and priming, suppresses PAD4 expression and NET release, and reduces production of matrix-degrading enzymes and osteoclastogenic factors. In this integrated model, PAD4-mediated NETosis inhibition represents a key node within a broader therapeutic network, rather than the sole initiating mechanism. Future studies using cell-type-specific PAD4 knockout models or selective inhibitors will help delineate the relative contribution of NETosis inhibition to catalpol’s overall anti-arthritic effects. Nevertheless, this multi-target activity profile may enhance therapeutic potential by concurrently addressing multiple facets of RA pathogenesis. In this integrated model, catalpol’s reduction of phospho-p65 and downstream cytokines (IL-1β, IL-6) aligns with its known pharmacology, likely acting as an upstream event that downregulates PAD4 along with other inflammatory mediators. This positions PAD4/NETosis inhibition not as an isolated target, but as a significant component within catalpol’s broader anti-inflammatory network. Further studies using cell-type-specific PAD4 knockouts or selective inhibitors will help clarify the relative contribution of NETosis inhibition to catalpol’s overall anti-arthritic effects. Nonetheless, its multi-target profile may enhance therapeutic potential by concurrently addressing multiple facets of RA pathogenesis.

This study has several limitations. Firstly, the absence of a positive drug control group restricts the interpretation of the results and their clinical relevance. Methotrexate and biological agents are established clinical standards for treating RA, with well-defined mechanisms and efficacy. A comparison with these positive drugs would provide a more direct evaluation of therapeutic effects of CAT and potential advantages. Without a positive control, it becomes challenging to directly compare catalpol’s efficacy to existing treatment options, limiting the assessment of its clinical application potential. Moreover, this study utilized the CIA mouse model, whereas RA’s pathological mechanisms are complex and heterogeneous. A single model may not adequately represent the disease’s full spectrum. Other models, such as transgenic or adjuvant-induced arthritis models, could better simulate different RA pathologies, such as self-antibody-mediated inflammation or T-cell-driven immune responses. Employing multiple models would offer a more comprehensive evaluation of catalpol’s therapeutic effects and applicability, as relying solely on the CIA model may not fully capture its mechanisms and limitations. It should be noted that our findings are derived from a single-sex (male) model, while clinical RA demonstrates significant sex disparities in prevalence and progression. Future studies incorporating female animal models and examining potential sex-dependent responses will be valuable to fully characterize catalpol’s therapeutic potential across biological contexts.

However, this study primarily focused on the role of catalpol in inhibiting NET formation through PAD4, establishing a novel anti-RA mechanism. Future investigations should consider a broader mechanistic scope to determine whether catalpol also operates through other pathways in RA treatment. Moreover, direct comparisons with more selective PAD4 inhibitors, such as GSK484, would better delineate the specificity and efficacy of catalpol’s PAD4 inhibitory activity. To strengthen the translational relevance of these findings, several important research directions should be pursued. First, validation in human-derived models, including neutrophils from RA patients or human synovial explants, will be critical for confirming clinical applicability. Second, employing genetic or highly selective pharmacological interventions targeting PAD4 could establish a clearer causal link between PAD4 inhibition and the therapeutic outcomes. While this study focused on PAD4 as the key driver of neutrophil NETosis, other isoforms of the PAD family are also expressed in relevant cell types. Although Cl-amidine, used as a reference inhibitor in this work, exhibits relatively high selectivity for PAD4, and our transcriptomic and Western blot analyses strongly support the involvement of PAD4, it remains to be determined whether catalpol—as a natural compound—may also affect other PAD isozymes such as PAD2. Future studies employing *in vitro* enzymatic activity assays or more selective genetic/pharmacological tools (e.g., isoform-specific inhibitors or knockout models) could help further clarify the selectivity of catalpol for PAD4 and thereby refine the understanding of its molecular target. Third, our study focused on the neutrophil-NET axis as a primary mechanism. However, RA pathogenesis involves a complex interplay of multiple cell types, including macrophages, fibroblast-like synoviocytes, and lymphocytes. While we observed downstream reductions in pro-inflammatory cytokines and tissue-destructive enzymes, the direct effect of catalpol on these other cell populations remains to be elucidated. Future work should investigate whether catalpol modulates macrophage polarization, FLS activation, or T-cell responses, which could contribute to its overall therapeutic effect.

## Data Availability

The datasets presented in this study can be found in online repositories. The names of the repository/repositories and accession number(s) can be found in the article/**Supplementary Material**.

## References

[1] Díaz-González F Hernández-Hernández MV. Rheumatoid arthritis. Med Clin. (2023) 161:533–42. doi: 10.1016/j.medcli.2023.07.014, PMID: 37567824

[2] Kao W Gu R Jia Y Wei X Fan H Harris J . A formyl peptide receptor agonist suppresses inflammation and bone damage in arthritis. Br J Pharmacol. (2014) 171:4087–96. doi: 10.1111/bph.12768, PMID: 24824742 PMC4243981

[3] Di Matteo A Bathon JM Emery P . Rheumatoid arthritis. Lancet (London England). (2023) 402. doi: 10.1016/s0140-6736(23)01525-8, PMID: 38240831

[4] Prasad P Verma S Surbhi, Ganguly NK Chaturvedi V Mittal SA . Rheumatoid arthritis: advances in treatment strategies. Mol Cell Biochem. (2023) 478:69–88. doi: 10.1007/s11010-022-04492-3, PMID: 35725992

[5] Pavlov-Dolijanovic S Bogojevic M Nozica-Radulovic T Radunovic G Mujovic N . Elderly-onset rheumatoid arthritis: characteristics and treatment options. Med (Kaunas Lithuania). (2023) 59(10):1878. doi: 10.3390/medicina59101878, PMID: 37893596 PMC10608066

[6] Wang W Zhou H Liu L . Side effects of methotrexate therapy for rheumatoid arthritis: A systematic review. Eur J Med Chem. (2018) 158:502–16. doi: 10.1016/j.ejmech.2018.09.027, PMID: 30243154

[7] Roubille C Richer V Starnino T McCourt C McFarlane A Fleming P . The effects of tumour necrosis factor inhibitors, methotrexate, non-steroidal anti-inflammatory drugs and corticosteroids on cardiovascular events in rheumatoid arthritis, psoriasis and psoriatic arthritis: a systematic review and meta-analysis. Ann Rheumatic Dis. (2015) 74:480–9. doi: 10.1136/annrheumdis-2014-206624, PMID: 25561362 PMC4345910

[8] Xie X Li F Li S Tian J Chen JW Du JF . Application of omics in predicting anti-TNF efficacy in rheumatoid arthritis. Clin Rheumatol. (2018) 37:13–23. doi: 10.1007/s10067-017-3639-0, PMID: 28600618

[9] Fawcett JW Fyhn M Jendelova P Kwok JCF Ruzicka J Sorg BA . The extracellular matrix and perineuronal nets in memory. Mol Psychiatry. (2022) 27:3192–203. doi: 10.1038/s41380-022-01634-3, PMID: 35760878 PMC9708575

[10] Aslanian-Kalkhoran L Mehdizadeh A Aghebati-Maleki L Danaii S Shahmohammadi-Farid S Yousefi M . The role of neutrophils and neutrophil extracellular traps (NETs) in stages, outcomes and pregnancy complications. J Reprod Immunol. (2024) 163:104237. doi: 10.1016/j.jri.2024.104237, PMID: 38503075

[11] de Souza FFL Schneider AH MaChado CC de Almeida SCL da Silva TA Cunha FQ . Synovial fluid survivin and NETs as independent biomarkers in rheumatoid arthritis. Clin Exp Rheumatol. (2023) 41:1473–9. doi: 10.55563/clinexprheumatol/flzpgb, PMID: 36441653

[12] Englert H Rangaswamy C Kullik GA Divivier M Göbel J Hermans-Borgmeyer I . Sepsis-induced NET formation requires MYD88 but is independent of GSDMD and PAD4. FASEB J. (2025) 39:e70301. doi: 10.1096/fj.202402514R, PMID: 39777764 PMC11707982

[13] Mao J Tan M Li J Liu C Hao J Zheng J . Neutrophil extracellular traps induce pyroptosis of rheumatoid arthritis fibroblast-like synoviocytes via the NF-κB/caspase 3/GSDME pathway. Inflammation. (2024) 47:921–38. doi: 10.1007/s10753-023-01951-x, PMID: 38133702

[14] Okamoto Y Devoe S Seto N Minarchick V Wilson T Rothfuss HM . Association of sputum neutrophil extracellular trap subsets with igA anti-citrullinated protein antibodies in subjects at risk for rheumatoid arthritis. Arthritis Rheumatol (Hoboken NJ). (2022) 74:38–48. doi: 10.1002/art.41948, PMID: 34369110 PMC8712364

[15] Wright HL Lyon M Chapman EA Moots RJ Edwards SW . Rheumatoid arthritis synovial fluid neutrophils drive inflammation through production of chemokines, reactive oxygen species, and neutrophil extracellular traps. Front Immunol. (2020) 11:584116. doi: 10.3389/fimmu.2020.584116, PMID: 33469455 PMC7813679

[16] Komatsu N Takayanagi H . Mechanisms of joint destruction in rheumatoid arthritis - immune cell-fibroblast-bone interactions. Nature reviews. Rheumatology. (2022) 18:415–29. doi: 10.1038/s41584-022-00793-5, PMID: 35705856

[17] Wu Z Ma D Yang H Gao J Zhang G Xu K . Fibroblast-like synoviocytes in rheumatoid arthritis: Surface markers and phenotypes. Int Immunopharmacol. (2021) 93:107392. doi: 10.1016/j.intimp.2021.107392, PMID: 33529910

[18] O’Neil LJ Oliveira CB Wang X Navarrete M Barrera-Vargas A Merayo-Chalico J . Neutrophil extracellular trap-associated carbamylation and histones trigger osteoclast formation in rheumatoid arthritis. Ann Rheumatic Dis. (2023) 82:630–8. doi: 10.1136/ard-2022-223568, PMID: 36737106 PMC11302494

[19] Fang Q Stehr AM Naschberger E Knopf J Herrmann M Stürzl M . No NETs no TIME: Crosstalk between neutrophil extracellular traps and the tumor immune microenvironment. Front Immunol. (2022) 13:1075260. doi: 10.3389/fimmu.2022.1075260, PMID: 36618417 PMC9816414

[20] Fischer V Haffner-Luntzer M . Interaction between bone and immune cells: Implications for postmenopausal osteoporosis. Semin Cell Dev Biol. (2021) 123:14–21. doi: 10.1016/j.semcdb.2021.05.014, PMID: 34024716

[21] Yap KH Yee GS Candasamy M Tan SC Md S Abdul Majeed AB . Catalpol ameliorates insulin sensitivity and mitochondrial respiration in skeletal muscle of type-2 diabetic mice through insulin signaling pathway and AMPK/SIRT1/PGC-1α/PPAR-γ Activation. Biomolecules. (2020) 10:1360. doi: 10.3390/biom10101360, PMID: 32987623 PMC7598587

[22] Bhattamisra SK Yap KH Rao V Choudhury H . Multiple biological effects of an iridoid glucoside, catalpol and its underlying molecular mechanisms. Biomolecules. (2019) 10(1):32. doi: 10.3390/biom10010032, PMID: 31878316 PMC7023090

[23] Zeng YF Wang R Bian Y Chen WS Peng L . Catalpol attenuates IL-1β Induced matrix catabolism, apoptosis and inflammation in rat chondrocytes and inhibits cartilage degeneration. Med Sci Monit. (2019) 25:6649–59. doi: 10.12659/msm.916209, PMID: 31484919 PMC6752111

[24] Wu B Dong Q Zhang Q Jin F Weng J . Protective effects of Catalpol to attenuate TNF- α and collagen-induced inflammation *in vitro* HFLS-RA cells and *in vivo* mice models for the treatment of rheumatoid arthritis. Clin Rheumatol. (2025) 44:1041–56. doi: 10.1007/s10067-024-07261-3, PMID: 39907970

[25] Di Y Zhang M Chen Y Sun R Shen M Tian F . Catalpol inhibits tregs-to-th17 cell transdifferentiation by up-regulating let-7g-5p to reduce STAT3 protein levels. Yonsei Med J. (2022) 63:56–65. doi: 10.3349/ymj.2022.63.1.56, PMID: 34913284 PMC8688372

[26] Khandpur R Carmona-Rivera C Vivekanandan-Giri A Gizinski A Yalavarthi S Knight JS . NETs are a source of citrullinated autoantigens and stimulate inflammatory responses in rheumatoid arthritis. Sci Transl Med. (2013) 5:178ra40. doi: 10.1126/scitranslmed.3005580, PMID: 23536012 PMC3727661

[27] Liu X Arfman T Wichapong K Reutelingsperger CPM Voorberg J Nicolaes GAF . PAD4 takes charge during neutrophil activation: Impact of PAD4 mediated NET formation on immune-mediated disease. J Thromb Haemost. (2021) 19:1607–17. doi: 10.1111/jth.15313, PMID: 33773016 PMC8360066

[28] Li B Cao X Ai G Liu Y Lv C Jin L . Interleukin-37 alleviates myocardial injury induced by coxsackievirus B3 via inhibiting neutrophil extracellular traps formation. Int Immunopharmacol. (2022) 113:109343. doi: 10.1016/j.intimp.2022.109343, PMID: 36308891

[29] Pang Y Zhao L Ji X Guo K Yin X . Analyses of Transcriptomics upon IL-1β-Stimulated Mouse Chondrocytes and the Protective Effect of Catalpol through the NOD2/NF-κB/MAPK Signaling Pathway. Mol (Basel Switzerland). (2023) 28(4):1606. doi: 10.3390/molecules28041606, PMID: 36838594 PMC9962284

[30] Aletaha D Smolen JS . Diagnosis and management of rheumatoid arthritis: A review. JAMA. (2018) 320:1360–72. doi: 10.1001/jama.2018.13103, PMID: 30285183

[31] Xiang M Yin M Xie S Shi L Nie W Shi B . The molecular mechanism of neutrophil extracellular traps and its role in bone and joint disease. Heliyon. (2023) 9:e22920. doi: 10.1016/j.heliyon.2023.e22920, PMID: 38076128 PMC10703630

[32] Di Matteo A Bathon JM Emery P . Rheumatoid arthritis. . Lancet. (2023) 402:2019–33. doi: 10.1016/s0140-6736(23)01525-8 38240831

[33] Warjukar PR Mohabey AV Jain PB Bandre GR . Decoding the correlation between inflammatory response marker interleukin-6 (IL-6) and C-reactive protein (CRP) with disease activity in rheumatoid arthritis. Cureus. (2024) 16:e62954. doi: 10.7759/cureus.62954, PMID: 39050325 PMC11265957

[34] VanSaun MN Matrisian LM . Matrix metalloproteinases and cellular motility in development and disease. *Birth Defects Res C Embryo Today: Reviews*. (2006) 78:69–79. doi: 10.1002/bdrc.20061, PMID: 16622849

[35] Mehana EE Khafaga AF El-Blehi SS . The role of matrix metalloproteinases in osteoarthritis pathogenesis: An updated review. Life Sci. (2019) 234:116786. doi: 10.1016/j.lfs.2019.116786, PMID: 31445934

[36] Lu R Wang YG Qu Y Wang SX Peng C You H . Dihydrocaffeic acid improves IL-1β-induced inflammation and cartilage degradation via inhibiting NF-κB and MAPK signalling pathways. Bone Joint Res. (2023) 12:259–73. doi: 10.1302/2046-3758.124.Bjr-2022-0384.R1, PMID: 37492935 PMC10076109

[37] Stojanovic SK Stamenkovic BN Cvetkovic JM Zivkovic VG Apostolovic MRA . Matrix metalloproteinase-9 level in synovial fluid-association with joint destruction in early rheumatoid arthritis. Med (Kaunas Lithuania). (2023) 59(1):167. doi: 10.3390/medicina59010167, PMID: 36676791 PMC9863294

[38] Gossiel F Ugur A Peel NFA Walsh JS Eastell R . The clinical utility of TRACP-5b to monitor anti-resorptive treatments of osteoporosis. Osteoporosis Int. (2022) 33:1357–63. doi: 10.1007/s00198-022-06311-3, PMID: 35102444

[39] Wen X Yi LZ Liu F Wei JH Xue Y . The role of cathepsin K in oral and maxillofacial disorders. Oral Dis. (2016) 22:109–15. doi: 10.1111/odi.12378, PMID: 26458004

[40] Chu C Wang X Chen F Yang C Shi L Xu W . Neutrophil extracellular traps aggravate intestinal epithelial necroptosis in ischaemia-reperfusion by regulating TLR4/RIPK3/FUNDC1-required mitophagy. Cell Prolif. (2024) 57:e13538. doi: 10.1111/cpr.13538, PMID: 37691112 PMC10771116

[41] Liu X Li T Chen H Yuan L Ao H . Role and intervention of PAD4 in NETs in acute respiratory distress syndrome. Respir Res. (2024) 25:63. doi: 10.1186/s12931-024-02676-7, PMID: 38291476 PMC10829387

[42] Biron BM Chung CS O’Brien XM Chen Y Reichner JS Ayala A . Cl-amidine prevents histone 3 citrullination and neutrophil extracellular trap formation, and improves survival in a murine sepsis model. J Innate Immun. (2017) 9:22–32. doi: 10.1159/000448808, PMID: 27622642 PMC5219946

[43] Suzuki M Ikari J Anazawa R Tanaka N Katsumata Y Shimada A . PAD4 deficiency improves bleomycin-induced neutrophil extracellular traps and fibrosis in mouse lung. Am J Respir Cell Mol Biol. (2020) 63:806–18. doi: 10.1165/rcmb.2019-0433OC, PMID: 32915635

[44] Knight JS Subramanian V O’Dell AA Yalavarthi S Zhao W Smith CK . Peptidylarginine deiminase inhibition disrupts NET formation and protects against kidney, skin and vascular disease in lupus-prone MRL/lpr mice. Ann Rheum Dis. (2015) 74:2199–206. doi: 10.1136/annrheumdis-2014-205365, PMID: 25104775 PMC4320672

[45] Smith MH Berman JR . What is rheumatoid arthritis? JAMA (2022) 327(12):1194. doi: 10.1001/jama.2022.0786, PMID: 35315883

[46] Kondo N Kuroda T Kobayashi D . Cytokine networks in the pathogenesis of rheumatoid arthritis. Int J Mol Sci. (2021) 22(20):10922. doi: 10.3390/ijms222010922, PMID: 34681582 PMC8539723

[47] Shapouri-Moghaddam A Mohammadian S Vazini H Taghadosi M Esmaeili SA Mardani F . Macrophage plasticity, polarization, and function in health and disease. J Cell Physiol. (2018) 233:6425–40. doi: 10.1002/jcp.26429, PMID: 29319160 PMC13482560

[48] Taylor PC Feist E Pope JE Nash P Sibilia J Caporali R . What have we learnt from the inhibition of IL-6 in RA and what are the clinical opportunities for patient outcomes? Ther Adv Musculoskeletal Dis. (2024) 16:1759720X241283340. doi: 10.1177/1759720x241283340, PMID: 39444594 PMC11497505

[49] Yang P Qian FY Zhang MF Xu AL Wang X Jiang BP . Th17 cell pathogenicity and plasticity in rheumatoid arthritis. J Leukocyte Biol. (2019) 106:1233–40. doi: 10.1002/jlb.4ru0619-197r, PMID: 31497905

[50] Bun M Kawano M Yamamoto G Sakata M Shimura K Toda A . G-CSF induces neutrophil extracellular traps formation and promotes ovarian cancer peritoneal dissemination. J Leukocyte Biol. (2024) 116:1157–68. doi: 10.1093/jleuko/qiae166, PMID: 39082070

[51] Tang C Jia F Wu M Wang Y Lu X Li J . Elastase-targeting biomimic nanoplatform for neurovascular remodeling by inhibiting NETosis mediated AlM2 inflammasome activation in ischemic stroke. J Controlled Release. (2024) 375:404–21. doi: 10.1016/j.jconrel.2024.09.026, PMID: 39288890

[52] Marcinkiewicz J Walczewska M . Neutrophils as sentinel cells of the immune system: A role of the MPO-halide-system in innate and adaptive immunity. Curr Med Chem. (2020) 27:2840–51. doi: 10.2174/0929867326666190819123300, PMID: 31424363

[53] Shen Z Gu J Jiang B Long H Li Z Chen C . Glycyrrhizin inhibits LPS-induced neutrophil-like release of NETs. Am J Trans Res. (2024) 16:5507–15. doi: 10.62347/larn2372, PMID: 39544807 PMC11558380

[54] Bonilha CS Veras FP Dos Santos Ramos A Gomes GF Rodrigues Lemes RM Arruda E . PAD4 inhibition impacts immune responses in SARS-CoV-2 infection. Mucosal Immunol. (2025) 18:861–73. doi: 10.1016/j.mucimm, PMID: 40258416

[55] Suzuki A Kochi Y Shoda H Seri Y Fujio K Sawada T . Decreased severity of experimental autoimmune arthritis in peptidylarginine deiminase type 4 knockout mice. BMC Musculoskelet Disord. (2016) 17:205. doi: 10.1186/s12891-016-1055-2, PMID: 27150598 PMC4858923

