# Supplementary material for: Catalpol mitigates rheumatoid arthritis by targeting neutrophil extracellular trap release
Source: Front Immunol. 2026 Mar 16;17:1763586. doi: 10.3389/fimmu.2026.1763586 (PMC13033488; doi:10.3389/fimmu.2026.1763586)
Supplement: Supplementary file 1 [file Table1.docx]

**1.Characterization of neutrophil isolation and optimal catalpol concentration for NETs inhibition**


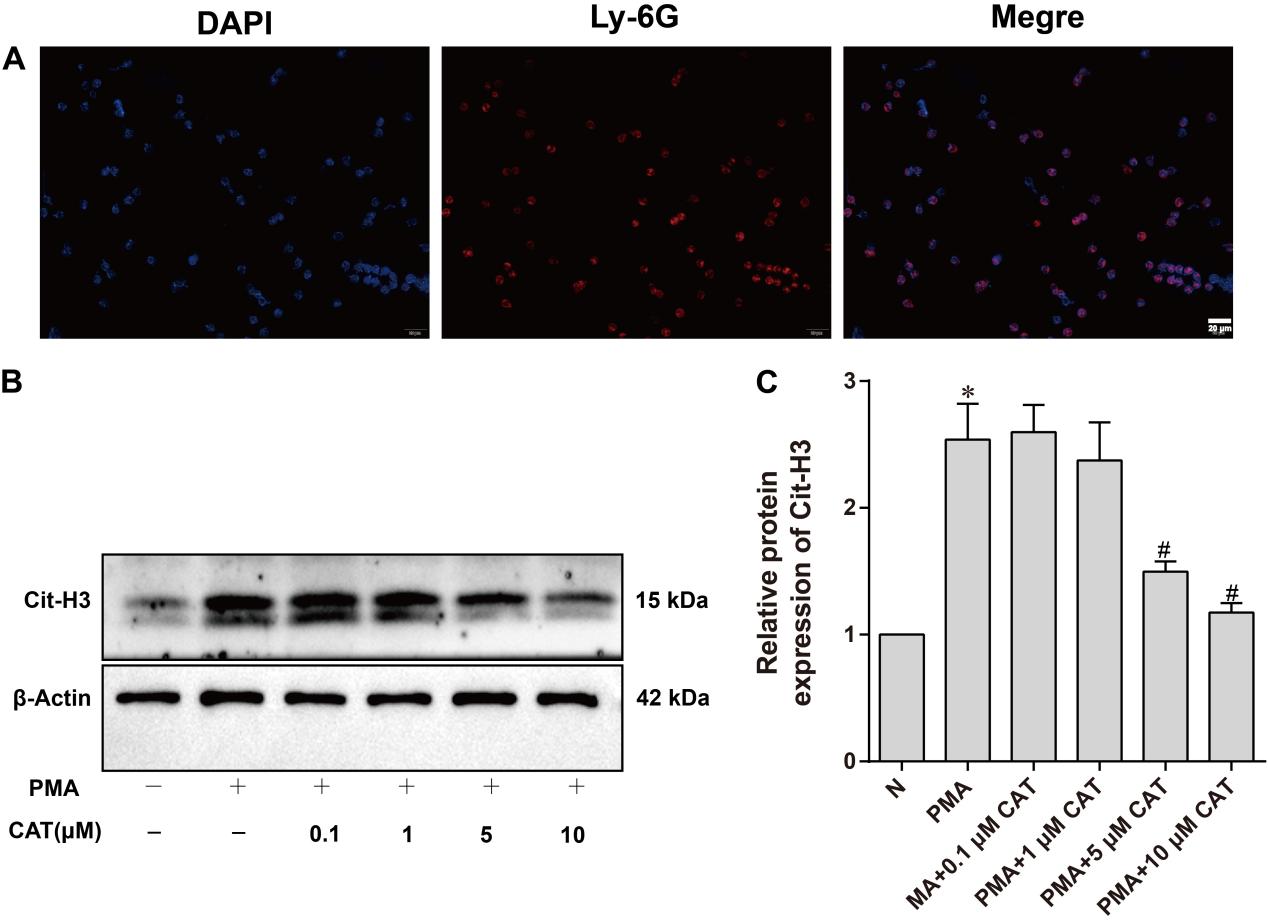


Figure 1. Immunofluorescence analysis of neutrophil purity and effect of catalpol on NETs formation. (A) Immunofluorescence staining for neutrophil identification. Representative images showing Ly-6G (red) and nuclear (DAPI, blue) staining. Scale bar: 20 μm. (B) Representative Western blot bands of Cit-H3 expression. (C) Quantitative analysis of Cit-H3 protein levels. Data are presented as mean ± SD from three independent experiments (n=3 per group). Statistical analysis was performed using Kruskal-Wallis test followed by Dunn's post hoc test (applied due to the limited sample size). **P* < 0.05 vs. control group (N); ^#^*P* < 0.05 vs. PMA-treated group.

The lymphocyte antigen 6 complex G locus (Ly-6G) is a surface marker highly specific for neutrophils. To validate the purity and specificity of the isolated neutrophils, we performed Ly-6G immunofluorescence staining. The results demonstrated that the isolated cells exhibited clear membrane-localized Ly-6G-positive signals. High-power microscopy revealed characteristic multi-lobed or horseshoe-shaped nuclei, confirming successful isolation of neutrophils with typical morphological features and high purity (Figure 1 A). To determine the optimal concentration of CAT for inhibiting neutrophil extracellular trap (NET) formation, we first evaluated its effect on neutrophil viability across a range of concentrations. Our data confirmed that catalpol did not adversely affect neutrophil viability at the tested concentrations (1-10 μM). Following this, we assessed the expression level of citrullinated histone H3 (Cit-H3) after treatment with various concentrations of CAT using Western blot analysis. The results (Figure 1B) demonstrated a concentration-dependent inhibition of Cit-H3 expression: inhibition was observed starting at 1 μM CAT, and significant suppression was achieved at 10 μM (P < 0.05). Although higher concentrations of CAT were not tested for their effect on Cit-H3 expression, based on the current experimental data and statistical analysis, we determined that 10 μM was the optimal concentration for significantly inhibiting NET formation (Figure 1 B).
