# Supplementary material for: Catalpol mitigates rheumatoid arthritis by targeting neutrophil extracellular trap release
Source: Front Immunol. 2026 Mar 16;17:1763586. doi: 10.3389/fimmu.2026.1763586 (PMC13033488; doi:10.3389/fimmu.2026.1763586)
Supplement: Supplementary file 2 [file Table2.docx]

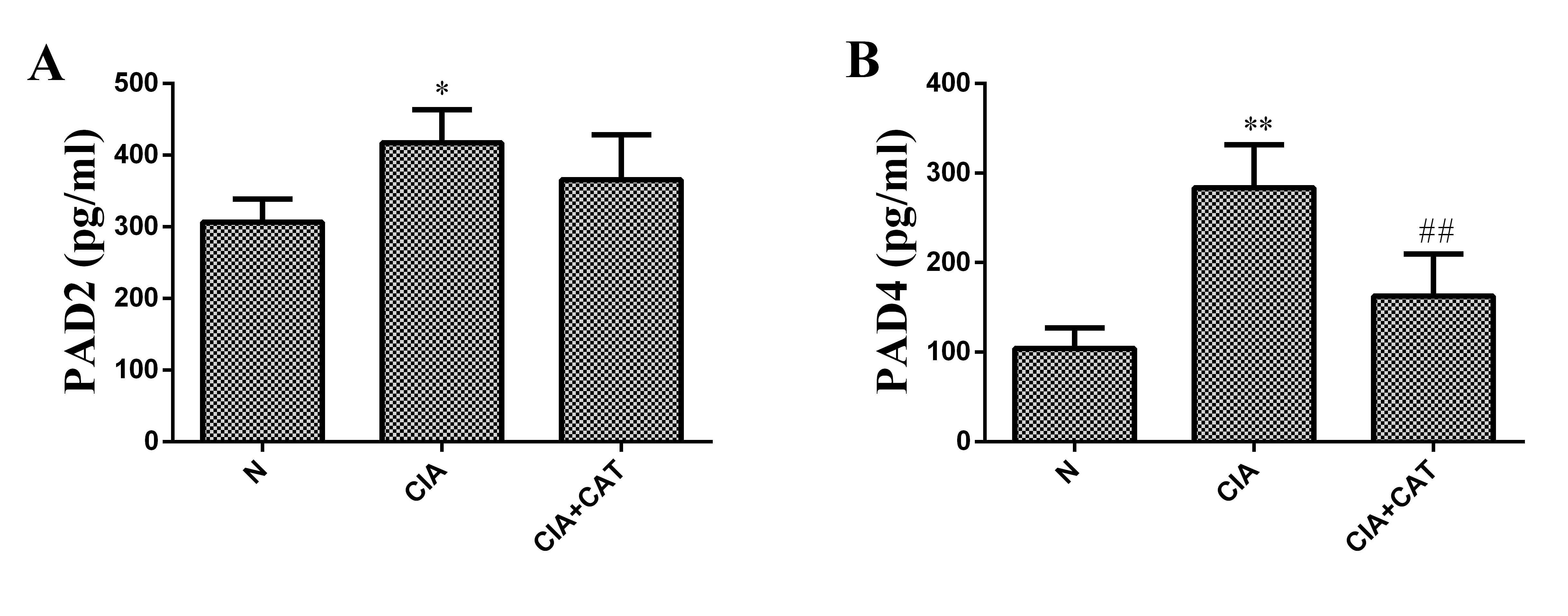


Supplementary Figure 2 Effects of CAT on PAD2 and PAD4 Levels in Joint Tissues of RA Mice

1. PAD2, (2) PAD4. Data are presented as mean ± SD from three independent experiments (n=3 per group). Statistical analysis was performed using Kruskal-Wallis test followed by Dunn's post hoc test (applied due to the limited sample size).**P* < 0.05, ***P* < 0.01 vs. N group; ^#^*P* < 0.05, ^##^*P* < 0.01 vs. CIA group (n=5 per group).

Both PAD4 and PAD2 protein levels were elevated in the CIA model group compared to the Normal group, consistent with the known involvement of both isozymes in RA pathogenesis. Catalpol treatment significantly reduced PAD4 levels in the CIA+CAT group compared to the CIA group. PAD2 levels also showed a decreasing trend following catalpol treatment, although the reduction was less pronounced than that observed for PAD4.

These findings suggest that while catalpol's therapeutic effect in CIA is associated with a significant and specific reduction in PAD4, a potential modulatory effect on PAD2 cannot be entirely ruled out. This is a valuable observation that aligns with the complexity of peptidylarginine deiminase biology in inflammation, as highlighted by the reviewer and the referenced literature on compensatory mechanisms.
