# Supplementary material for: Catalpol mitigates rheumatoid arthritis by targeting neutrophil extracellular trap release
Source: Front Immunol. 2026 Mar 16;17:1763586. doi: 10.3389/fimmu.2026.1763586 (PMC13033488; doi:10.3389/fimmu.2026.1763586)
Supplement: Supplementary file 3 [file Table3.docx]

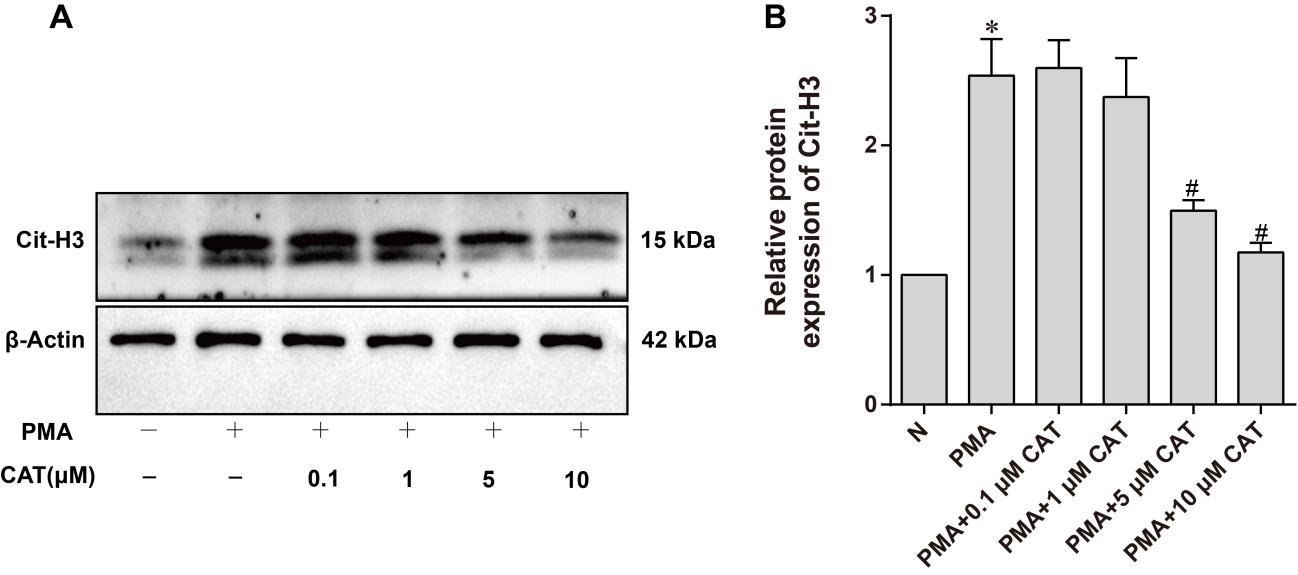


Figure S3 Screening of catalpol concentration for significant inhibition of NETs formation. (A) Representative Western blot images. (B) Quantitative analysis of relative Cit-H3 protein expression. Data are presented as mean ± SD. **P* < 0.05 vs. N group; #*P* < 0.05 vs. PMA group (n = 3 per group).

To determine the optimal concentration of CAT for inhibiting NET formation, the expression levels of Cit-H3 were assessed by Western blot following treatment with various concentrations of CAT. As shown in Figure S3, Cit-H3 expression decreased progressively with increasing CAT concentrations. A downward trend in Cit-H3 expression was observed starting at 1 µM CAT, and at 10 µM, Cit-H3 protein levels were significantly reduced and reached the lowest level among the tested concentrations. Although the effect of higher CAT concentrations on Cit-H3 expression was not examined, based on the current experimental data and statistical analysis, 10 µM was identified as the appropriate concentration for CAT to significantly inhibit NETs formation.
